# Depicting the dynamic transcriptional and epigenetic landscape of testis development in pubertal Simmental cattle

**DOI:** 10.1186/s40104-026-01406-x

**Published:** 2026-05-18

**Authors:** Junmei Zhang, Xin Qi, Wenxuan Zhao, Shijie Yuan, Na Hai, Leqian Yu, Jinlian Hua, Dawei Yu, Yulei Wei

**Affiliations:** 1 State Key Laboratory of Animal Biotech Breeding, Frontiers Science Center for Molecular Design Breeding, College of Biological Sciences, China Agricultural University, Beijing, 100193 China; 2 State Key Laboratory of Stem Cell and Reproductive Biology, Institute of Zoology, Chinese Academy of Sciences, Beijing, 100101 China; 3 Institute for Stem Cell and Regeneration, Chinese Academy of Sciences, Beijing, 100101 China; 4 State Key Laboratory of Animal Biotech Breeding, Institute of Animal Sciences, Chinese Academy of Agricultural Sciences (CAAS), Beijing, 100193 China; 5 College of Veterinary Medicine, Shaanxi Centre of Stem Cells Engineering & Technology, Northwest A&F University, Yangling, Shaanxi 712100 China; 6 National Germplasm Center of Domestic Animal Resources, Institute of Animal Science, Chinese Academy of Agricultural Sciences, Beijing, 100193 China

**Keywords:** Single-cell RNA sequencing, Single-nucleus ATAC sequencing, Simmental cattle, Spermatogenesis

## Abstract

**Background:**

The reproductive development of Simmental cattle, a vital breed for global beef production, remains poorly understood at the molecular and cellular levels. A systematic analysis of the regulatory mechanisms governing germ cell fate transitions during testicular development is essential for advancing breeding efficiency and reproductive technologies in cattle.

**Results:**

Using integrated single-cell RNA sequencing (scRNA-seq) and single-nucleus ATAC sequencing (sNucATAC-seq) on testicular tissues from Simmental cattle across postnatal (PN), prepubertal (PP), and pubertal (PUB) stages, we identified core transcriptional regulators—including E2F1, BCLAF1, and YY1—that govern germ cell fate transitions. Several signaling pathways, such as TGF-β, MAPK, ErbB, and AMPK, were found to participate in spermatogenic processes. Sertoli cells were classified into three functional subtypes: Stage 1 is associated with the transition from spermatogonial stem cells (SSCs) to differentiating spermatogonia (Diff-SPG), Stage 2 correlates with development from Diff-SPG to spermatocytes (SPC), and Stage 3 coincides with the transformation from SPC to spermatids (SPT). Cross-species comparative transcriptomics with humans, pigs, and mice revealed conserved pathways in germ cell development, with E2F1 notably conserved during the SSC-to-Diff-SPG transition.

**Conclusions:**

This study deciphers the regulatory network controlling germ cell fate transitions during bovine testicular development. The identification of conserved regulators and pathways provides novel insights into spermatogenesis and supports the development of strategies to overcome meiotic barriers in stem cell systems. These findings pave the way for precision breeding and advanced reproductive technologies in mammals.

**Supplementary Information:**

The online version contains supplementary material available at 10.1186/s40104-026-01406-x.

## Background

Spermatogenesis constitutes a sophisticated developmental cascade within the mammalian testis, wherein SSCs undergo mitotic proliferation to enlarge the progenitor pool, then meiotic division to ultimately form haploid spermatozoa [1–4]. This precisely regulated process not only guarantees genomic integrity through error-free chromosome segregation but also executes coordinated cell state transition involving stage-specific morphological remodeling, organelle reorganization, and functional specialization [5–7].

The journey of spermatogenesis commences with SSCs, which arise from fetal precursors known as prospermatogonia or gonocytes. These progenitor cells progress through several developmental milestones, diverging into self-renewing SSCs and Diff-SPG. Housed on the basement membrane of the seminiferous tubules, SSCs act as the foundational source for the ongoing production of sperm [8, 9]. The Diff-SPG further undergo mitotic divisions and develop into type B SPG, which then enter meiosis, forming primary SPC [10]. These primary SPC undergo the first meiotic division to produce secondary SPC, which quickly complete a second division to generate haploid round SPT. The SPT then undergo spermiogenesis, ultimately leading to the release of mature sperm into the lumen of the seminiferous tubules [11–13].

Besides germ cells, there exist various somatic cells in the testis. Sertoli cells offer structural and nutritional aid, uphold the blood-testis barrier, and assist in sperm release [14]. Peritubular myoid cells help maintain tubular structure and contractility, and Leydig cells produce testosterone, which is critical for germ cell development and function [15]. In summary, spermatogenesis involves complex interactions among multiple cell types. The intricate crosstalk between germ cells and somatic cells regulates not only germ cell proliferation, differentiation, and apoptosis but also sperm morphogenesis and maturation processes [1].

In commercial beef cattle operations, the efficiency of spermatogenesis serves as a key factor for breeding success, directly influencing semen quality parameters, fertilization capacity, and subsequent herd productivity [16, 17]. Understanding the molecular mechanisms governing bovine spermatogenesis, with particular emphasis on the signaling networks that regulate the balance between SSC self-renewal and differentiation, is critical for identifying key control points at specific developmental stages. Such mechanistic insights will empower the development of targeted reproductive biotechnologies for precise germline engineering, thereby overcoming the efficiency limitations of conventional labor-intensive breeding techniques and enhancing genetic selection to improve breeding outcomes. Indeed, some groups have provided crucial insights into the molecular mechanisms underlying bovine spermatogenesis. Nevertheless, compared to other livestock species such as pigs, systematic research on spermatogenesis in beef cattle remains relatively limited, and the molecular regulatory networks as well as key mechanisms involved require further investigation. Only two studies have provided insights into germ cell-related genes in Angus [18] and Simmental cattle [19] during pubertal transitions. In Angus, scRNA-seq of testicular cells from prepubertal and pubertal stages revealed distinct cellular clusters and identified stage-specific markers. In Simmental cattle, direct RNA sequencing identified testicular weight-associated differentially expressed genes and revealed heightened alternative splicing activity during sexual maturation. However, these investigations exhibit significant limitations: 1) Both studies employed a single sequencing method, which failed to capture the comprehensive view of gene expression regulation; 2) They simply profiled germ cell gene expression without comprehensive exploration of core mechanisms governing germ cell fate determination; 3) They failed to investigate somatic cells, resulting in an incomplete understanding of spermatogenic regulation; 4) The lack of multi-species comparative analysis in these two studies limits a comprehensive understanding of the mechanisms underlying spermatogenesis, given that conserved regulatory mechanisms may exist across different species.

To systematically investigate the core scientific question of spermatogenesis mechanisms in beef cattle, we implemented a systematic research strategy. By expanding the sample size to include testicular tissues from seven Simmental cattle (seven independent biological replicates), we obtained complete testicular tissue samples across three critical developmental stages for the first time: postnatal, prepubertal, and pubertal. This established a temporal dynamic research model for spermatogenesis, achieving precise coverage of the complete cycle of germ cell development. Using scRNA-seq and sNucATAC-seq, we comprehensively investigated the spermatogenesis process during Simmental cattle growth, and systematically analyzed stage-specific transcriptional landscapes and vital marker genes across spermatogenesis. Furthermore, we focused on three key somatic cell populations: Sertoli cells, Leydig cells, and peritubular myoid cells and studied their maturation processes. Through cell–cell interaction analysis, we delineated their intricate regulatory networks with germ cells, elucidating mechanisms that coordinate germ cell proliferation, differentiation, and sperm morphogenesis. These findings provide a comprehensive framework for understanding the complex biology of bovine spermatogenesis. Additionally, comparative analyses across testicular cell lineages of humans, pigs, mice, and cattle have unveiled evolutionarily conserved regulatory modules, providing valuable insights for reproductive biology research.

## Methods

### Testicular sample collection

Testicular tissue samples were collected from Simmental cattle with fully traceable pedigrees and birth records, all obtained from Tongliao Jingyuan Breeding Bull Co., Ltd. This ensured accurate age determination and clear genetic background information for all subjects. Based on these criteria, healthy individuals at three key developmental stages—postnatal (PN), prepubertal (PP), and pubertal (PUB)—were systematically selected. A total of seven individuals were sampled across these stages: two at PN (one for scRNA-seq and one for sNucATAC-seq), three at PP (two for scRNA-seq and one for sNucATAC-seq), and two at PUB (one for scRNA-seq and one for sNucATAC-seq). All samples designated for sNucATAC-seq profiling were supported by stringent, fully documented records of genetic background and age (Tables S1 and S2). All procedures strictly adhered to the Guidelines for the Care and Use of Laboratory Animals issued by the Beijing Association for Laboratory Animal Science, with ethical approval granted by the Institutional Animal Care and Use Committee of the Beijing Institute of Animal Science and Veterinary Medicine, Chinese Academy of Agricultural Sciences. Following veterinary-administered anesthesia using an approved protocol, testes were aseptically excised using sterile surgical techniques. Excised tissues underwent sequential processing: 1) thorough perfusion with Penicillin-Streptomycin solution (P1400, Solarbio, Beijing, China) to eliminate biological contaminants; 2) immersion in a specialized tissue preservation medium (130-100-008, Miltenyi Biotec, Bergisch Gladbach, Germany). All specimens were maintained at 4 °C during transport and processed within 6 h post-collection for downstream research.

### Isolation and culture of primary bovine testicular Sertoli cells

Fresh bovine testicular tissue was minced and transferred into DMEM/F12 medium (10-092-CV, Corning, New York, USA) containing 1 mg/mL Collagenase IV (C8160, Solarbio, Beijing, China) and 1 mg/mL DNase I (D8071, Solarbio, Beijing, China). The mixture was digested at 37 °C on an orbital shaker (80 r/min) for 15–20 min to loosen the seminiferous tubule structure. The digestate was filtered through a 100-μm cell strainer. Seminiferous tubule fragments retained on the filter were collected and thoroughly rinsed with DPBS (P1020, Solarbio, Beijing, China). The collected tubule fragments were then transferred into a solution containing 0.25% Trypsin-EDTA (25200072, Thermo Fisher Scientific, Waltham, USA) and a small amount of DNase I, and digested at 37 °C for 15 min with intermittent gentle pipetting until cell clusters dispersed. Digestion was terminated by adding medium supplemented with 10% fetal bovine serum (FBS, 100-ES, Vistech, Auckland, New Zealand). The cell suspension was sequentially filtered through 100-μm and 40-μm cell strainers to obtain a single-cell suspension.

The cell suspension was seeded onto culture plates pre-coated with Gelatin (G7041-500G, Sigma-Aldrich, St. Louis, USA) and incubated at 37 °C in a 5% CO_2_ incubator. Sertoli cells typically adhered rapidly within 4 h, while most germ cells adhered more slowly or remained in suspension. After 24 h of culture, non-adherent cells were further removed by gentle medium change. The adherent primary Sertoli cells were cultured in DMEM (C11995500BT, Gibco, Waltham, USA) supplemented with 10% FBS, with the medium changed every 2–3 d.

### Isolation and culture of primary bovine skin cells

The skin tissue was cut into small fragments, which were evenly placed onto the bottom of a culture dish. A small amount of DMEM containing 10% FBS was then carefully added, and the culture was maintained at 37 °C. Once cells had migrated out from the tissue explants and reached confluency, they were subcultured.

### Preparation of testicular tissue for scRNA-seq and sNucATAC-seq

Testicular tissues from Simmental cattle were collected at specific developmental stages for RNA sequencing. For scRNA-seq, tissues were collected from animals at PN, PP1 and PP2, PUB. For sNucATAC-seq, tissues were collected from animals at PN, PP, and PUB. The tissues were minced and placed into 1.5-mL EP tubes labeled accordingly. Each tube was treated with 2 mg/mL of a mixture of Collagenase II (C8150, Solarbio, Beijing, China) and Collagenase IV (C8160, Solarbio, Beijing, China) and incubated in a water bath at 37 °C for 40 min, with pipetting every 10 min to ensure thorough digestion. Following digestion, the samples were centrifuged at 500 × *g* to remove the supernatant, and the pellet was resuspended in 1 mL of DMEM (10-013-CV, Corning, New York, USA), supplemented with 2% FBS (200-ES, Vistech, New Zealand). The cell suspension was then filtered through a 70-μm filter (352350, Corning, New York, USA) to remove cell clumps and washed with DMEM. The samples were centrifuged again at 300 × *g* for 5 min to remove impurities. Red blood cells were lysed using a red blood cell lysis buffer (C521, Magen, Guangzhou, China) to purify the cell population. The final pellet was resuspended in 1 × PBS (P1020, Solarbio, Beijing, China) containing 0.04% bovine serum albumin (BSA, 199899, MP Biomedicals, Santa Ana, USA), and the samples were prepared for single-cell sequencing.

### Construction and sequencing of single-cell transcriptomic libraries

Under the conditions of cell viability exceeding 85% and aggregation rate below 5%, the water-in-oil droplet library preparation was performed using microfluidic technology. The cell concentration was initially adjusted to 700–1,200 cells/μL. During library construction, individual Gel Beads and single cells were co-encapsulated within oil droplets to form Gel Bead-In-Emulsions (GEMs). Subsequently, in the GEM reaction system, cellular lysis was conducted to release mRNA, which was then reverse-transcribed into cDNA using Poly-dT primers. The synthesized cDNA underwent amplification followed by library construction. Quality control procedures included: initial quantification using Qubit 3.0 Fluorometer, determination of insert fragment size distribution through Agilent 2100 Bioanalyzer analysis, and measurement of effective library concentration (≥ 10 nmol/L) via StepOnePlus Real-Time PCR System. Qualified libraries meeting all quality parameters were subsequently subjected to sequencing on the Illumina platform employing the paired-end 150 (PE150) strategy.

### Histological staining and immunostaining of testicular tissue

Testicular tissue samples from Simmental cattle at appropriate developmental stages were fixed in 4% paraformaldehyde (P1110, Solarbio, Beijing, China) for 24 h at 4 °C. Subsequently, the samples underwent gradient ethanol dehydration, clarification with an environmentally friendly clarifying agent (XH81100, G-clone, Beijing, China), and tissue embedding and sectioning. After the sections were dried, they were subjected to hematoxylin and eosin (H&E) staining and immunofluorescence staining, respectively.

For H&E staining, the sections were first immersed in hematoxylin (ZLI-9610, ZSGB-BIO, Beijing, China) stain for 10 min, then rinsed with distilled water for 5 min. They were treated with 1% hydrochloric acid alcohol differentiation solution for 30 s, followed by rinsing with tap water for 3 min to enhance the clarity of nuclear staining. The sections were then immersed in eosin stain for 2 min to impart a red color to the cytoplasm. After rehydration and clarification, the sections were mounted with neutral gum.

For immunofluorescence staining, the sections were first dewaxed and rehydrated, then washed with distilled water and subjected to antigen retrieval. They were permeabilized with 0.5% Triton X-100/PBS solution for 30 min, followed by blocking with 5% BSA blocking solution for 1 h at room temperature to reduce nonspecific binding. Primary antibodies (Table 1) were added and incubated overnight at 4 °C. The sections were gently washed three times with PBS, each for 10 min. Fluorescently labeled secondary antibodies (Table 1) were added and incubated for 1 h at room temperature. The sections were then gently washed three times with PBS, each for 10 min. DAPI solution was added for staining at room temperature for 5 min, followed by washing with PBS three times, each for 10 min. The sections were mounted with Anti-fluorescent decay agent (E-IR-R119, Elabscience, Wuhan, China). The H&E-stained sections were imaged using a panoramic tissue cell analyzer (Leica Aperio VESA8, Leica, Wetzlar, Germany). The immunofluorescence-stained sections were imaged using a confocal laser scanning microscope (A1 confocal system, Nikon, Tokyo, Japan).

**Table 1:** Antibodies used in this study

Antibodies	Source	Identifier
DDX4	Abcam	ab27591
UCHL1	Huabio	EM1701-86
KI67	Bd	550609
MLH1	Huabio	ET1604-41
PNA	Invitrogen	L21409
SOX9	Sigma	AB5535
VIM	Huabio	EM0401
CYP11A1	CST	14217S
CD34	Abcam	ab81289
DAZL	Abcam	Ab34139
ELAVL2	Boster	A06194-2
DMRT1	Boster	PB0609
Donkey anti-Mouse IgG AF488	Thermo	A32766
Goat anti-Rabbit IgG AF488	Thermo	A11008
Donkey anti-Mouse IgG AF555	Thermo	A32773
HRP-conjugated Goat Anti-Rabbit IgG (H + L)	ZSGB-BIO	ZB-5301

For immunohistochemistry, tissue sections were dewaxed, rehydrated, and subjected to antigen retrieval. Endogenous peroxidase activity was quenched by incubation in 3% hydrogen peroxide for 15 min at room temperature. The sections were then blocked with 5% BSA (36101ES60, Yeasen, Shanghai, China) for 1 h and subsequently incubated with primary antibodies (Table 1) overnight at 4 °C. Following three washes with PBS (5 min each), the sections were incubated with HRP-conjugated secondary antibodies (Table 1) for 1 h at room temperature. After an additional PBS wash, signals were developed using a 3,3′-diaminobenzidine (DAB) substrate kit (ZLI‑9018, ZSGB‑BIO, Beijing, China), with the reaction progress monitored under a microscope. The chromogenic reaction was terminated by rinsing with deionized water. Nuclei were counterstained with hematoxylin (ZLI‑9610, ZSGB‑BIO, Beijing, China). Finally, the sections were dehydrated through a graded ethanol series, cleared with an environmentally friendly clearing agent (XH81100, G-clone, Beijing, China), and mounted with a permanent mounting medium. Stained sections were examined and imaged using an upright light microscope (OPTOME series, Zeiss, Jena, Germany).

### Lentiviral packaging and transduction of bovine SSCs

Multiple shRNAs targeting genes of interest were designed and cloned into the pLKO.1 vector (Table 2). Lentiviral particles were produced in HEK293T cells. The cells were seeded into 10-cm dishes 24 h prior to transfection to achieve 70%–80% confluency. The shRNA lentiviral transfer plasmid, packaging plasmid psPAX2, and envelope plasmid pMD2.G were combined at a mass ratio of 5:3.25:1.75 and co-transfected using PEI MW40000 (40816, YEASEN, Shanghai, China). After incubation at room temperature for 15 min, the complexes were added dropwise to the cultures. The medium was replaced with fresh complete medium the following day. Viral supernatants were harvested at 48 h and 72 h post-transfection, concentrated using a lentivirus concentration reagent (ES-7011, ECOTOP, Beijing, China), and stored at −80 °C. For stable knockdown, immortalized bovine SSCs were transduced. Cells were seeded in 6-well plates 24 h before transduction to reach 30%–50% confluency. The medium was replaced with fresh medium containing the concentrated viral stock and 8 µg/mL polybrene (TR-1003, Sigma-Aldrich, St. Louis, USA). Following overnight incubation at 37 °C under 5% CO_2_, the virus-containing medium was removed, and the cells were washed twice with DPBS before the addition of fresh medium. At 48 h post-transduction, puromycin (HY-B1743, MCE, Monmouth Junction, USA) was added to a final concentration of 2 µg/mL to select for transduced cells. After 2 days of selection, cells were harvested for analysis of target gene expression.

**Table 2:** List of primers and shRNAs used in this study

Genes	Application	Sequences (5′→3′)
*E2F1*	shRNA	ccggCTCACTCCTGGAGCACGTGAActcgagTTCACGTGCTCCAGGAGTGAGtttttg
		aattcaaaaaCTCACTCCTGGAGCACGTGAActcgagTTCACGTGCTCCAGGAGTGAG
*YY1*	shRNA	ccggCGACGGTTGTAATAAGAAGTTctcgagAACTTCTTATTACAACCGTCGtttttg
		aattcaaaaaCGACGGTTGTAATAAGAAGTTctcgagAACTTCTTATTACAACCGTCG
*BCLAF1*	shRNA	ccggGAGGTTCACTTCGTATCAGAActcgagTTCTGATACGAAGTGAACCTCtttttg
		aattcaaaaaGAGGTTCACTTCGTATCAGAActcgagTTCTGATACGAAGTGAACCTC
*DMRT1*	RT-PCR	Forward: CAGCCGTCTCTGTTTCCGTA
		Reverse: CCGAGTTTTTCATCTGCCACT
*LIN28B*	RT-PCR	Forward: AGGCCCCGGAGGTTCTTC
		Reverse: TCTTCTCCACCACCTTTGCC
*NT5DC1*	RT-PCR	Forward: TGATTCAGTTTCCGGGCTGG
		Reverse: AGTCCAGGGGTAATTCTGCT
*ELAVL2*	RT-PCR	Forward: TAGCGTTGGACTTTGGGGTC
		Reverse: TGGAGTTGCCGTCTTTCTCC
*STAG3*	RT-PCR	Forward: TCTGGATCAGATGCTTCCCAGAA
		Reverse: TTAACAAGACAAAAGCCTGCTCCT
*MRPS25*	RT-PCR	Forward: CTGCAGTACCTCAGTCAGGG
		Reverse: GTTGAAAAACACGAATTTCCTGGC
*DAZL*	RT-PCR	Forward: TACATGCAGCCTCCAACCAT
		Reverse: TGGTTGTATAAGCCGGAGGA
*ADGRG1*	RT-PCR	Forward: GGACTGGGAAGAAGCTGACC
		Reverse: CGAGACATCACTGCCTGGAT
*MERTK*	RT-PCR	Forward: CGTCCATCTATCCGTCGGTC
		Reverse: ATCTCAGCAATAGCTCGCGT
*P3H2*	RT-PCR	Forward: CCTACTATCGAGTCGGCGAA
		Reverse: CGCTGTCACCCAGTAGACTC
*FRMPD1*	RT-PCR	Forward: CCATTGGCTTCGGGCTTAGG
		Reverse: AGCAGAGAATTACCCAGACTGC
*MAP3K21*	RT-PCR	Forward: AGCCCAACGTCAAGAAGAGG
		Reverse: TTTGTGCTGGAAGTCTGAAGGT
*TOX*	RT-PCR	Forward: TGTGGGATGGTTTGGGTGAA
		Reverse: AGGCTCGCTGTAGCTCTTGG
*E2F1*	RT-PCR	Forward: CCACCCAGGAAAAGGTGTGA
		Reverse: CGAGCGGCTCAGTAATTCCA
*YY1*	RT-PCR	Forward: CTGGAGGGATACCTGGCATT
		Reverse: CTGAACATCTTTGTGCAGCCTTTA
*BCLAF1*	RT-PCR	Forward: CTTCGGCGTGTCAGGAATTTA
		Reverse: AGCGACCCATTTCTTTTCTCCT
*PCNA*	RT-PCR	Forward: AACTTGGCCATGGGCGTGAA
		Reverse: TGCCAACGTGTCCGCGTTAT
*CDK2*	RT-PCR	Forward: TCCGCCTGGACACTGAGACA
		Reverse: GTGAGCGCAGAGGCATCCAT
*CCND1*	RT-PCR	Forward: CGTACCCCGATGCCAACCTC
		Reverse: CGCAGACCTCCAGCATCCAG
*MKI67*	RT-PCR	Forward: CAACACTCTGAAGAAGCCGC
		Reverse: GAAGTCCCTCGGTTTGCTGA
*UCHL1*	RT-PCR	Forward: CGAGGATGGGTCTGTTCTGA
		Reverse: GGCATCCGTCCATCAAGTTC
*OCT4*	RT-PCR	Forward: AACGAGAATCTGCAGGAGATATG
		Reverse: TCTCACTCGGTTCTCGATACT
*GAPDH*	RT-PCR	Forward: CCTGCCCGTTCGACAGATA
		Reverse: GGCGACGATGTCCACTTTG
*SOX9*	RT-PCR	Forward: GCTCTGGAGACTGCTGAAC
		Reverse: CCGTTCTTCACCGACTTCC

### Quantitative reverse transcription PCR (qRT-PCR)

Total RNA was extracted using Trizol (R4801-02, Huayun Biotechnology, Guangzhou, China). The concentration and purity of the RNA were measured using a NanoPhotometer N50 (IMPLEN, Munich, Germany). Subsequently, the RNA was reverse-transcribed into complementary DNA (cDNA) using the Evo M-MLV RT Mix Kit (AG11728, AGbio, Hunan, China). qRT-PCR was performed using the ChamQ Universal SYBR qPCR Master Mix (Q711-02, Vazyme, Nanjing, China) on a QuantStudio™ 5 Real-Time PCR System (Applied Biosystems, Foster City, CA, USA). The sequences of the primers used are listed in Table 2. *GAPDH* was used as the housekeeping gene for normalization. The relative expression levels of target genes were calculated using the 2^−ΔΔCt^ method.

### Single-cell RNA sequencing data analysis and integration

The raw sequencing data was processed utilizing cellranger software (v7.2.0), which is provided by 10x Genomics (https://www.10xgenomics.com/). The latest reference genome and gene annotation files for Simmental cattle (Bos_taurus.ARS-UCD1.3) were obtained from Ensembl (https://www.ensembl.org/) and were prepared using the mkgtf and mkref tools of cellranger, employing the default settings. Subsequently, the gene expression count matrix for downstream analysis was generated through the cellranger count function with default parameters.

We applied DoubletFinder package (v2.0.3) to each sample individually to remove doublets. The workflow was as follows: based on the normalized data and clustering results, we optimized the parameters to determine the optimal pK value. The expected doublet rate was calculated using an empirical formula (8 × 10⁻⁶ per cell) and then adjusted by the proportion of homotypic doublets. Finally, with pN set to 0.25 and the optimized parameters, doublets were identified and removed, retaining high-confidence singlets for downstream analysis. Filtering of these samples was conducted individually using the Seurat package (v4.3.0) (https://satijalab.org/seurat/), with the following criteria: cells with fewer than approximately 400 expressed genes, more than approximately 11,000 UMIs, or more than 35% of reads mapping to the mitochondrial genome were removed. After adding their sample information, the samples were merged into a seurat object.

Multiple samples were normalized with NormalizeData and scaled with the merged features which were generated by SelectIntegrationFeatures, using the FindIntegrationAnchors function with a specified parameter (reduction ="﻿cca", k.filter = 400) to identify common anchors between four datasets. Subsequent to this step, the data integration process was carried out using the IntegrateData function based on the identified anchors. After integration, the ScaleData and RunPCA functions were applied to the merged dataset. Furthermore, the cell distribution was visualized using the RunUMAP function with non-default parameter: ‘dims = 1:35’. AddModuleScore was used to calculate the pathway score between different cell groups.

### Single-nucleus ATAC sequencing data analysis and integration

The raw sequencing data was processed utilizing cellranger-atac software (v2.1.0), which is provided by 10x Genomics (https://www.10xgenomics.com/). The same reference files were prepared using mkref tools, employing the default settings. Subsequently, the files for downstream analysis were generated through the cellranger-atac count function with default parameters.

The data was processed using the Seurat and Signac (v1.11.0) packages. Based on the calculated common peaks, the assay data of ATAC was created by using the FeatureMatrix and CreateChromatinAssay methods. Meanwhile, the GTF file was transformed into the EnsDb format annotation data by using the ensDbFromGtf and GetGRangesFromEnsDb methods. The genomic positions were mapped and annotated with it. Low-quality cells were removed by filtering. All samples were subjected to quality control. Key metrics included transcription start site (TSS) enrichment score and fraction of reads in peaks (FRiP). All samples had a TSS enrichment score greater than 4 and an FRiP value above 0.2, indicating high-quality chromatin accessibility data. Samples were merged with RunTFIDF and RunSVD. Furthermore, the cell distribution was visualized using the RunUMAP function with non-default parameter (reduction = "lsi", ‘dims = 2:50’).

We calculated gene activity using GeneActivity and depicted peak accessibility with CoveragePlot.

### Identification of developmental-related genes and transcription factors (TFs)

The identification of genes of interest was conducted through a comprehensive multi-step analytical approach. Initially, differentially expressed genes (DEGs) across distinct cell types were identified using the default parameters of the FindAllMarkers function. These identified genes served as signature genes representative of each cell type. Following this, functional enrichment analysis was performed on these signature genes utilizing the enrichGO and enrichKEGG functions from the ClusterProfiler (v4.10.0). The objective of this enrichment analysis was to find functional pathways that were conserved across different cell types, with the genes involved in these pathways hypothesized to act as key regulators of transitions between cell types.

Additionally, comparative differential expression analysis was executed between the same cell types across different samples to identify genes that exhibited consistent or unique expression patterns throughout developmental samples. These genes were proposed to be useful for mediating the cellular changes.

### Single-cell regulatory network

Simmental cattle genes were translated into their corresponding human orthologs utilizing the biomaRt (v2.58.0). Subsequently, Single-Cell Regulatory Network Inference and Clustering (SCENIC) analysis was conducted utilizing the pySCENIC (v0.12.1) based on the human ortholog-transformed expression data. The regulon specificity score (RSS) was computed using the calcRSS function, and the results were visualized through the plotRSS function.

### Cell trajectory analysis

The R package monocle2 (v2.26.0) was used for reconstructing the cell differentiation trajectory. The monocle2 object was formed using the newCellDataSet function with the count matrix from the Seurat object and DEGs were calculated using differentialGeneTest. Subsequently, the pseudotime trajectory was established through the reduceDimension function (with parameter "DDRTree") and the orderCells function. Finally, the visualization of the pseudotime trajectory was achieved using the plot_cell_trajectory function.

The R package monocle3 (v1.3.5) was used for calculating the pseudotime of single-cell RNA data. The monocle3 object was formed using the new_cell_data_set function with the count matrix from the Seurat object. The monocle3 object was processed using the preprocess_cds function, incorporating the UMAP reduction information from Seurat. Subsequently, the pseudotime trajectory was established through the learn_graph and order_cells functions. The visualization of the pseudotime trajectory was achieved using the plot_cells function. Additionally, the trajectory-associated genes and their modules were identified using graph_test and find_gene_modules.

### Soft clustering of transcriptomic data

AverageExpression was used to create pseudo-bulk gene expression profiles categorized by cell type, which were then analyzed with Mfuzz (v2.62.0) to identify gene expression clusters.

### Protein–protein interaction network

The STRING database (https://string-db.org/) was used to create the protein–protein interaction network for the selected genes. Then, the Cytoscape (v3.8.0) software, along with the iRegulon and MCODE plugins, was employed to analyze this network. In particular, MCODE identified subnetworks within the interaction network, and iRegulon predicted TFs that might regulate the genes present in that subnetwork.

### Cross-species analysis

Single-cell datasets from human and pig were normalized using the NormalizeData function independently. The FindIntegrationAnchors function was utilized with its default settings, and the IntegrateData function was subsequently employed to integrate the species. The resulting integrated data were subsequently processed with the ScaleData and RunPCA functions. Furthermore, the cell distribution was visualized using the RunUMAP function.

The cellular distribution of the mouse samples was obtained through the application of NormalizeData, ScaleData, RunPCA, and RunUMAP functions without CCA integration. We determined the overall cell types and germ cell types in three species by using specific markers, and then employed Monocle3 to reconstruct the developmental trajectories of their germ cells. Afterward, we used CellAlign (v0.1.0) to compare the progression of these trajectories among four species (human, pig, cattle and mouse), and applied SAMap (v1.0.15) to analyze the cellular similarities between their cell types.

### Cell–cell communication

Simmental cattle genes were translated into their corresponding human orthologs utilizing the biomaRt (v2.58.0). Subsequently, the CellChat package (v1.6.1) was employed, using the CellChatDB.human database to infer cell–cell communication networks through functions such as computeCommunProb and computeCommunProbPathway to evaluate ligand-receptor interactions within various signaling pathways. To visualize the interactions, the netVisual_circle and subsetCommunication functions were implemented.

### Quantification and statistical analysis

All experiments were performed with at least three independent biological replicates. Statistical analysis was conducted using two-way analysis of variance (ANOVA) in GraphPad Prism (version 10.1.2). *P* value < 0.05 was indicated as "*", *P* value < 0.01 was indicated as "**", *P* value < 0.001 was indicated as "***", *P* value < 0.0001 was indicated as "****".

## Results

### Identification and analysis of cell types in the testes of Simmental cattle

**Fig. 1:**
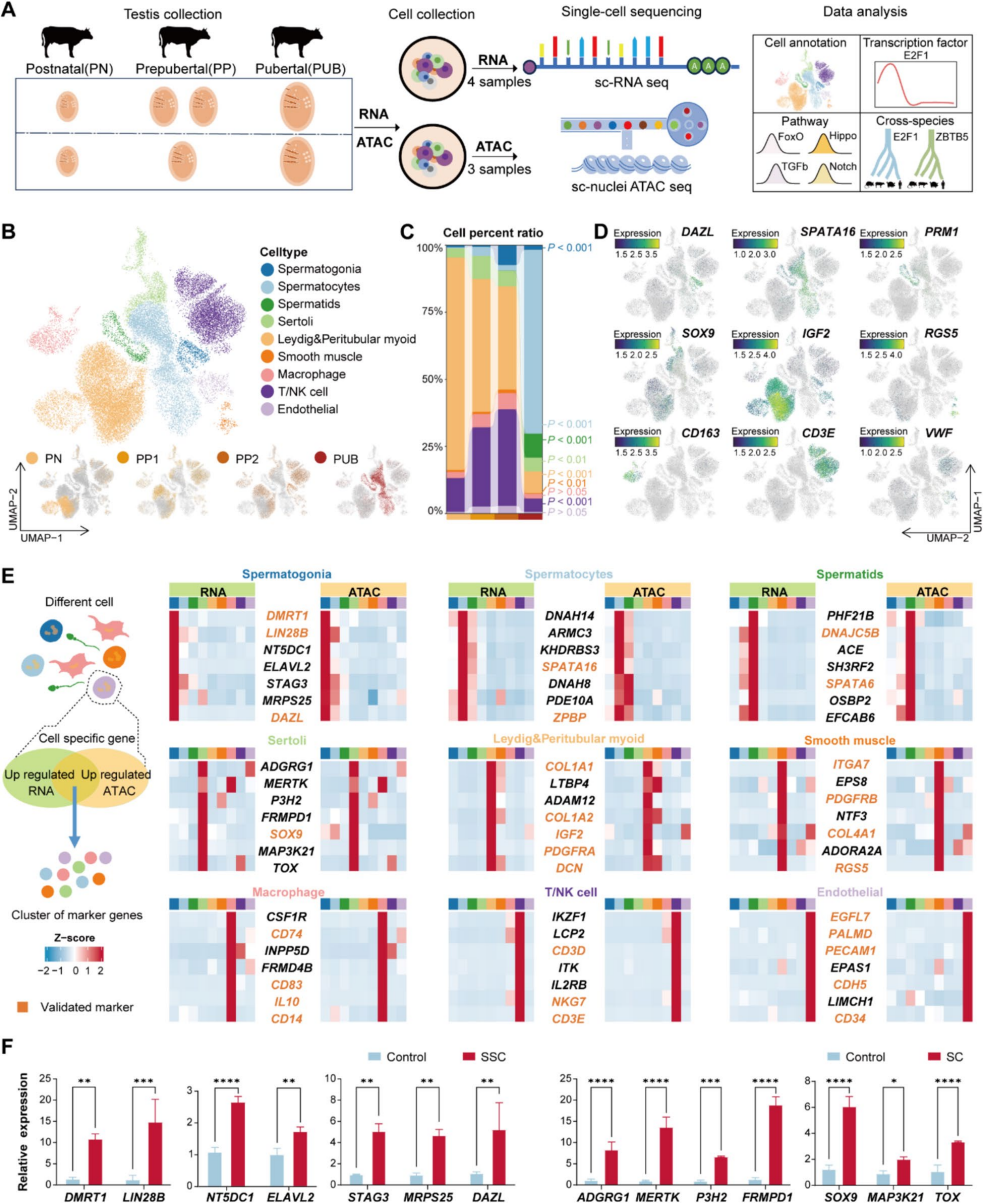
scRNA-seq and sNucATAC-seq of developing Simmental cattle testis. **A** Schematic diagram of the experimental workflow. **B** Uniform manifold approximation and projection (UMAP) visualization based on RNA levels shows the testicular cell types of Simmental cattle across PN, PP1, PP2 and PUB developmental stages. **C** Cell proportions of testicular cell types across developmental stages. *P*-values were calculated using the Cochran-Armitage trend test across the four developmental time points (PN, PP1, PP2, and PUB). **D** Expression of selected markers projected onto UMAP. **E** Identification of cell-type-specific genes by intersecting markers from both RNA and ATAC levels, with a heatmap displaying expression of 7 marker genes in testicular cell types at both levels. **F** Analysis of the relative expression levels of genes in bovine Sertoli cells and SSCs. The light blue bars in the figure represent the control group (bovine skin cells), while the red bars represent the experimental groups (bovine Sertoli cells and SSCs)

To delineate testicular developmental processes in Simmental cattle, we collected testicular tissues via castration at three critical stages: postnatal (PN), prepubertal (PP), and pubertal (PUB) (Fig. S1A). Histological analyses revealed significant morphological and compositional changes in seminiferous tubules: in PN testes, SPG were observed within the seminiferous tubules. By the PP stage, both SPG and SPC were identified, accompanied by an expansion of the tubular lumen. At the PUB stage, multiple germ cell types, including elongated SPT, became evident (Fig. S1B). To investigate the developmental trajectory, testicular tissues from multiple developmental stages were subjected to scRNA-seq and sNucATAC-seq. For scRNA-seq, one sample from the PN stage, two independent samples (designated PP1 and PP2) from the PP stage, and one from the PUB stage were included. For sNucATAC-seq, one sample from each of the PN, PP, and PUB stages was analyzed (Fig. 1A). We first conducted standard quality-control (QC) procedures on both the RNA and ATAC sequencing datasets, including doublet removal, to ensure data accuracy and reliability (Fig. S2A–S2C). After QC, a total of 32,878 high-quality cells were retained for the RNA-seq dataset and 40,099 cells for the ATAC-seq dataset. Then, we conducted UMAP analysis at both RNA and ATAC levels, and identified nine major cell types based on known specific cell markers [4, 7, 20–22]. Additionally, the distribution of samples was also displayed on the UMAP plot (Fig. 1B, Fig. S2E, S2F and Table S3). The credibility of the dataset was demonstrated through the specific expression patterns of marker genes in both spermatogenic and somatic cell types, with germ cells encompassed SPG (*DAZL*^+^), SPC (*SPATA16*^+^), and SPT (*PRM1*^+^), and somatic cells included Sertoli cells (*SOX9*^+^), Leydig cells and peritubular myoid cells (*IGF2*^+^), smooth muscle cells (*RGS5*^+^), macrophages (*CD163*^+^), T/NK cells (*CD3E*^+^), and endothelial cells (*VWF*^+^) (Fig. 1D). Based on the scRNA-seq data across four developmental stages, we observed dynamic and stage-specific shifts in the composition of testicular cell types. Germ cells showed a substantial increase in proportional abundance with developmental time. SPC was rare at PN (0.37%) but became the dominant population by PUB (68.53%), while SPT first appeared at PP2 (0.08%) and expanded to 8.96% at PUB. In contrast, most somatic cell types exhibited a progressive decrease in relative proportion. Leydig and peritubular myoid cells, which constituted the majority at PN (79.17%), markedly declined to 8.12% by PUB. Similarly, Sertoli cells decreased from 3.46% at PN to 5.09% at PUB. Smooth muscle cells remained relatively stable across stages, maintaining a low proportion. Immune cells also showed dynamic changes: T/NK cells increased from 12.54% at PN to a peak of 36% at PP2, then declined to 4.99% at PUB. Macrophages gradually increased from 2.28% at PN to 6.04% at PP2, then decreased to 1.72% by PUB. Endothelial cells comprised 0.61%–2.84% of the population across stages (Fig. 1C and Table S3). To identify novel and specific marker genes for testicular cell types in Simmental cattle, we conducted a comprehensive analysis of cell clustering at both the RNA and ATAC levels. Based on transcriptome profiles, we performed differential expression analysis to identify genes significantly upregulated in each cluster, which allowed us to pinpoint RNA-level marker genes for distinct cell types, including SPG, SPC, SPT, Sertoli cells, Leydig cells, and other interstitial components. Simultaneously, using sNucATAC-seq data, we analyzed chromatin accessibility patterns and identified regions with enriched accessibility within promoter and enhancer elements of key cell identity genes. By integrating dual-omics data, we cross-validated and refined a robust set of cell-type-specific markers, improving the accuracy of cell type annotation. To visually summarize these findings, the 7 marker genes for each cell type were visualized using a heatmap (Fig. 1E). Previous studies have identified *ELAVL2* as a testis-enriched factor that exhibits preferential expression in human and murine SSCs [23]. Similarly, *DMRT1* is a well-established critical marker for SPG, with evolutionarily conserved expression patterns across multiple mammalian species, including pigs [24] and dairy goats [25]. Both genes were enriched in our SPG cells. Consistently, *ZPBP* has been previously identified as a human testis-specific gene [26, 27], while *SPATA16* shows enriched expression in diplotene SPC. *DNAJC5B* [28] and *SPATA6* [29], which served as a bridge gene expressed within elongating SPT, and essential for the proper assembly of the segmented columns and capitulum during sperm connecting piece formation, respectively, were enriched in our SPT. Separately, *SOX9* is crucial for maintaining testicular integrity in Sertoli cells, functioning through the regulation of structural protein expression and the suppression of premature apoptosis [30]. *PDGFRA* expression was detected in Leydig cells and peritubular myoid cells; previous studies in mice have shown that *PDGFRA* deficiency impairs Leydig cells differentiation [31]. *RGS5* [32], *CD74* [25], *NKG7* [27], and *PECAM1* [27] expressions were visualized in smooth muscle cells, macrophages, T/NK and endothelial cells, respectively (Fig. S2G). The high concordance with previously reported markers attests to the robustness and accuracy of our dataset. Beyond known markers, our multi-omics approach enabled the discovery of several novel candidate marker genes exhibiting cell-type-specific expression patterns (Fig. 1E). These include *NT5DC1* and *STAG3* for SPG; *DNAH14* and *ARMC3* for SPC; *PHF21B* and *SH3RF2* for SPT; *ADGRG1* and *MERTK* for Sertoli cells; as well as *LTBP4* and *ADAM12* for Leydig and peritubular myoid cells. Additionally, we identified novel markers associated with other somatic compartments: *EPS8* (smooth muscle cells), *CSF1R* (macrophages), *IKZF1* (T/NK cells) and *EPAS1* (endothelial cells). To functionally characterize these markers, we validated the expression of key genes. Their transcription in SSCs and Sertoli cells was confirmed by qRT-PCR, while immunofluorescence and immunohistochemistry analyses further demonstrated the localized expression of proteins such as DAZL, DMRT1, and ELAVL2 within SPG (Fig. 1F and Fig. S2D). Together, these findings not only refine the molecular signatures of testicular cell types in Simmental cattle but also provide a valuable resource and a set of candidate targets for future investigations into spermatogenesis and testicular pathogenesis.

### Core transcription factors for stage-specific spermatogenesis in Simmental cattle

**Fig. 2:**
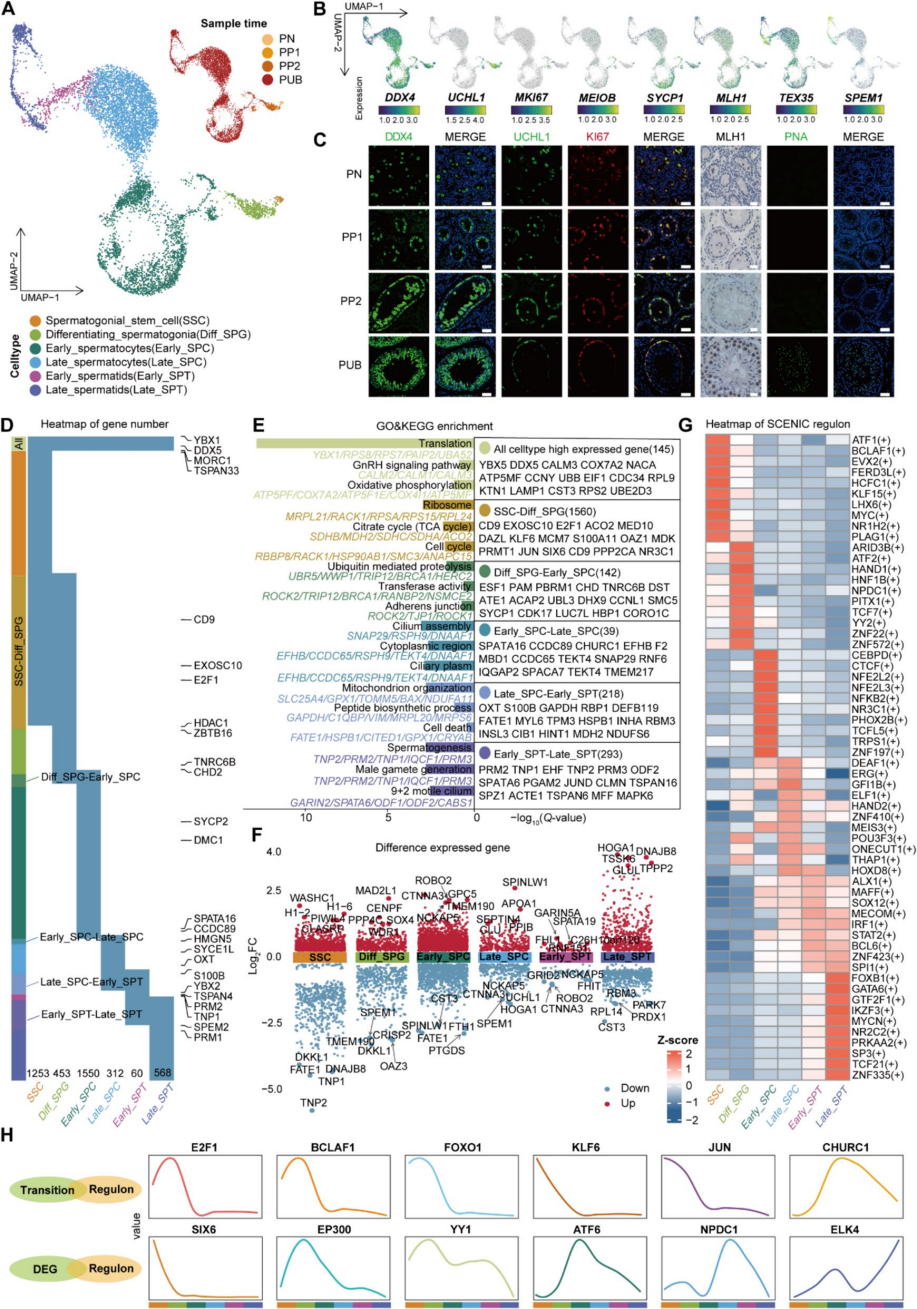
Coordinated gene-transcriptional regulation reveals stage-specific key regulators during spermatogenesis in Simmental cattle.** A** UMAP visualization based on RNA levels illustrates the germ cells of Simmental cattle across PN, PP1, PP2 and PUB developmental stages. **B** Expression of selected markers projected onto UMAP. **C** DDX4 staining demonstrates the localization of germ cells, UCHL1 and KI67 co-staining indicates the localization of Diff-SPG, MLH1 staining indicates the localization of SPC, and PNA staining reveals the localization of SPT. Scale bar: 50 μm. **D** Heatmap visualization of unique and shared genes across various developmental stages of germ cells. **E** Genes and signaling pathways involved in the fate transition of various germ cell types during different developmental stages of testis. **F** Volcano plot illustrating significantly upregulated or downregulated genes across various germ cell types. Differential expression was determined by comparing each cell type against all remaining cell types. **G** Heatmap displaying cell-type-specific regulon activity identified via SCENIC analysis. Color represents scaled regulon activity. **H** Genes associated with fate transitions of different types of germ cells at various developmental stages were obtained. 12 genes were found by taking the intersection of Fig. 2D and G

We employed integrated scRNA-seq and sNucATAC-seq to perform high-resolution clustering of *DDX4*^+^ germ cells, systematically delineating spermatogenesis in Simmental cattle from SPG to SPT. Leveraging conserved marker profiles, we defined six molecular sub-clusters (Fig. 2A, B and Table S4): *UCHL1*^+^ SSCs [33], *MKI67*^+^ Diff-SPG [34], *MEIOB*^+^/*SYCP1*^+^ Early-SPC [35–37], *MLH1*^+^ Late-SPC [38], *TEX35*^+^ Early-SPT [27], and *SPEM1*^+^ Late-SPT [27, 39]. Compositional analysis across stages indicated that the SSC proportion progressively declined with age. The proportion of Diff-SPG exhibited a trajectory characterized by an initial rise followed by a gradual decline, peaking in PP1 samples. Early-SPC was first identified at PN, with Late-SPC and early-SPT concomitantly emerging at PP2. Late-SPT was abundantly observed at PUB, marking the completion of the full spermatogenic cycle coinciding with sexual maturity (Fig. S3A and S3B). The sequencing data were further validated through immunofluorescence and immunohistochemistry analyses. DDX4-positive cell quantification established age-dependent germ cell population dynamics in testicular tissues. Immunofluorescence co-localization studies with UCHL1 and KI67 confirmed active SPG differentiation beginning at PN. MLH1 immunodetection confirmed its expression in both SPG and SPC. Peanut agglutinin (PNA^+^) cellular detection verified the presence of mature spermatozoa by PUB, confirming the functional completion of spermatogenesis (Fig. 2C).

To delineate changes in gene expression during spermatogenesis, we utilized the FindAllMarkers function to identify genes associated with this process, which identified 1,253 specific genes for SSCs, 453 for Diff-SPG, 1,550 for Early-SPC, 312 for Late-SPC, 60 for Early-SPT, and 568 for Late-SPT, with some genes reported previously (Fig. 2D and Table S4). We also discovered 145 genes that were expressed throughout spermatogenesis, along with 1,560 genes shared between SSCs and Diff-SPG, 142 between Diff-SPG and Early-SPC, 39 between Early-SPC and Late-SPC, 218 between Late-SPC and Early-SPT, and 293 between Early-SPT and Late-SPT (Fig. 2E and Table S4). These findings allowed us to explore their associated Gene Ontology (GO) terms and Kyoto Encyclopedia of Genes and Genomes (KEGG) pathways. As anticipated, biological processes such as "Cell cycle" were significantly enriched during SSC differentiation. Processes such as "Ubiquitin-mediated proteolysis" were significantly enriched during the transition from Diff-SPG to Early-SPC, "Cilium assembly" was enriched during the transition from Early-SPC to Late-SPC, and "Mitochondrial organization" was enriched during the transition from Late-SPC to Early-SPT (Fig. 2E). To visually represent the distinctive gene expression characteristics of different germ cell types, as well as the specific expression patterns of genes at critical transition points during germ cell fate changes, the distribution and expression changes of significantly upregulated and downregulated genes throughout this process were displayed (Fig. 2F and Fig. S3C). Signaling pathways including WNT, AMPK, FOXO and TGF-β were enriched during the transition from SSCs to Diff-SPG. The "ERAD signaling pathway" and others were enriched during the transition from Diff-SPG to Early-SPC, while the "ErbB signaling pathway" and additional pathways were enriched during the transition from Early-SPC to Late-SPC (Fig. S3D, Table S4 and Table S6).To further uncover the core regulatory factors across distinct germ cell types and systematically decipher their transcriptional regulatory networks, we employed the SCENIC analysis approach to identify the complete set of active regulons among all germ cell populations. The activity patterns of the 10 key regulons in different germ cell clusters were clearly visualized using a heatmap (Fig. 2G and Table S4). Through intersection analysis of both germ cell type-specific genes and those involved in germ cell fate transition with transcription factors specific to each germ cell population, we identified a set of key regulators, including E2F1, BCLAF1, FOXO1, KLF6, JUN and CHURC1, that were co-enriched in both transcriptional activity and target gene expression during germ cell fate transition. Furthermore, we revealed additional factors specifically co-enriched within unique germ cell types, such as SIX6, EP300, YY1, ATF6, NPDC1 and ELK4 (Fig. 2H). Interestingly, E2F1, BCLAF1 and FOXO1 showed high expression in Diff-SPG, while CHURC1 played key roles during the fate transition of SPC. In contrast, JUN, KLF6 and SIX6 showed low expression in Diff-SPG, and EP300 was highly expressed in Diff-SPG. Furthermore, this study also revealed that YY1 is highly expressed in SPG. Previous research has indicated that *YY1* can regulate the expression of spliceosome-related genes, broadly affecting the efficiency and accuracy of mRNA splicing in spermatogonial cells, thereby contributing to the maintenance of gene expression homeostasis in the process of spermatogenesis. These findings further suggest that *YY1* may perform stage-specific functions in spermatogonial cells [40, 41]. Additionally, ATF6 functions primarily in Early-SPC, NPDC1 exerts its main role in Late-SPC, while ELK4 is predominantly involved in regulating the process of spermatogenesis (Fig. 2H). To further validate the functions of these key transcription factors, we performed functional perturbation experiments targeting *E2F1*, *BCLAF1*, and *YY1* using a lentivirus-mediated shRNA knockdown system and assessed changes in the expression of relevant proliferation and stemness-related genes by qRT-PCR (Fig. S4A). The results showed that knockdown of *YY1* led to downregulation of both proliferation markers (e.g., *PCNA*, *CDK2*, *CCND1*, *MKI67*) and stemness markers (e.g., *OCT4*, *UCHL1*). This observation aligns with the hypothesized role of *YY1* in maintaining spermatogonial homeostasis and proliferative capacity, providing functional support for its stage-specific actions. Similarly, knockdown of *E2F1* and *BCLAF1* produced comparable phenotypic effects, further confirming the critical roles of these factors in regulating spermatogonial cell fate.

### Key genes and signaling pathways in germ cell fate transition

**Fig. 3:**
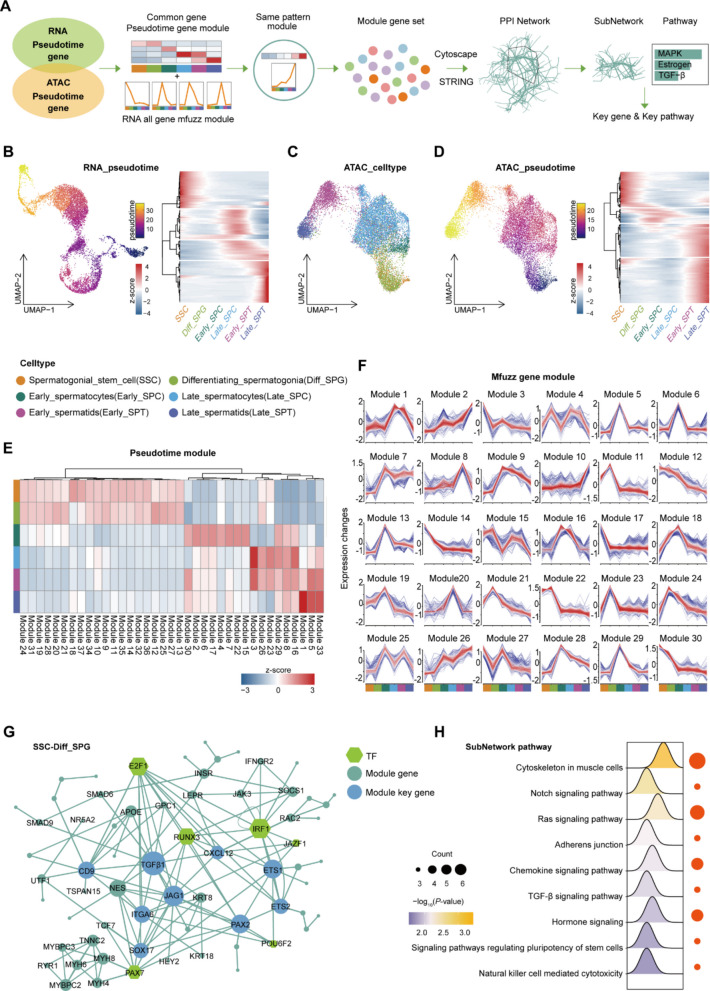
Core genes and pathways in germ cell fate transition of Simmental cattle. **A** Schematic representation of the workflow for obtaining germ cell fate transition genes and pathways: intersecting multi-omic trajectory-associated genes to identify expression modules, matching these with global modules at the RNA level, and constructing protein–protein interaction networks from the resulting overlapping genes to extract core subnetworks and biological functions. **B** RNA-level pseudotemporal trajectory analysis of germ cells. **C** UMAP visualization of germ cell clustering at the ATAC level. **D** ATAC-level pseudotemporal trajectory analysis of germ cells. **E** Gene modules obtained by integrating RNA and ATAC germ cell trajectory-related genes. **F** Mfuzz clustering analysis of gene expression dynamics in germ cells. **G** Protein–protein interaction network during the SSC-Diff-SPG developmental stage (Hexagon: TF; Green circle: module genes of network; Blue circle: module’s key genes of network). **H** Significantly active signaling pathways in the SSC-Diff-SPG stage

To identify key regulators of germ cell fate determination, we performed an integrated multi-omics trajectory analysis. Pseudotime ordering of germ cells was constructed using RNA-seq data, and further refined with ATAC-seq-based stratification (Fig. 3A–D and Fig. S4B). This integrated approach revealed 35 groups of gene modules with distinct expression trends along the developmental trajectory (Fig. 3E and Table S5). In parallel, Mfuzz analysis of all RNA expression data identified 30 co-expression modules (Fig. 3F and Table S5). By aligning the pseudotime and Mfuzz modules, we pinpointed modules with conserved dynamic patterns. Protein–protein interaction networks were then constructed using Cytoscape and the STRING database. Subnetworks corresponding to specific pseudotime windows were extracted and analyzed in the context of known biological pathways. This analysis identified stage-specific transcriptional regulators: E2F1, RUNX3, IRF1, and PAX7 were linked to the SPG window, with enrichment in Notch, Ras, and TGF-β signaling pathways. The SPC window was characterized by activity of GATA1, EGR1, and RUNX3, associated with ErbB, GnRH, and Hippo signaling. The transition from SPC to SPT involved BDP1, HIC1, MAX, BRF1, and THRB, with enrichment for terms like "Spermatogenesis" and "Sperm flagellum" (Fig. 3G, H, Fig. S4C–S4E, and Table S5). These findings not only corroborate known regulatory mechanisms in germ cell differentiation but also provide novel molecular insights into fate determination.

### Sertoli cells orchestrate spermatogenesis via specific and interrelated signaling pathways

**Fig. 4:**
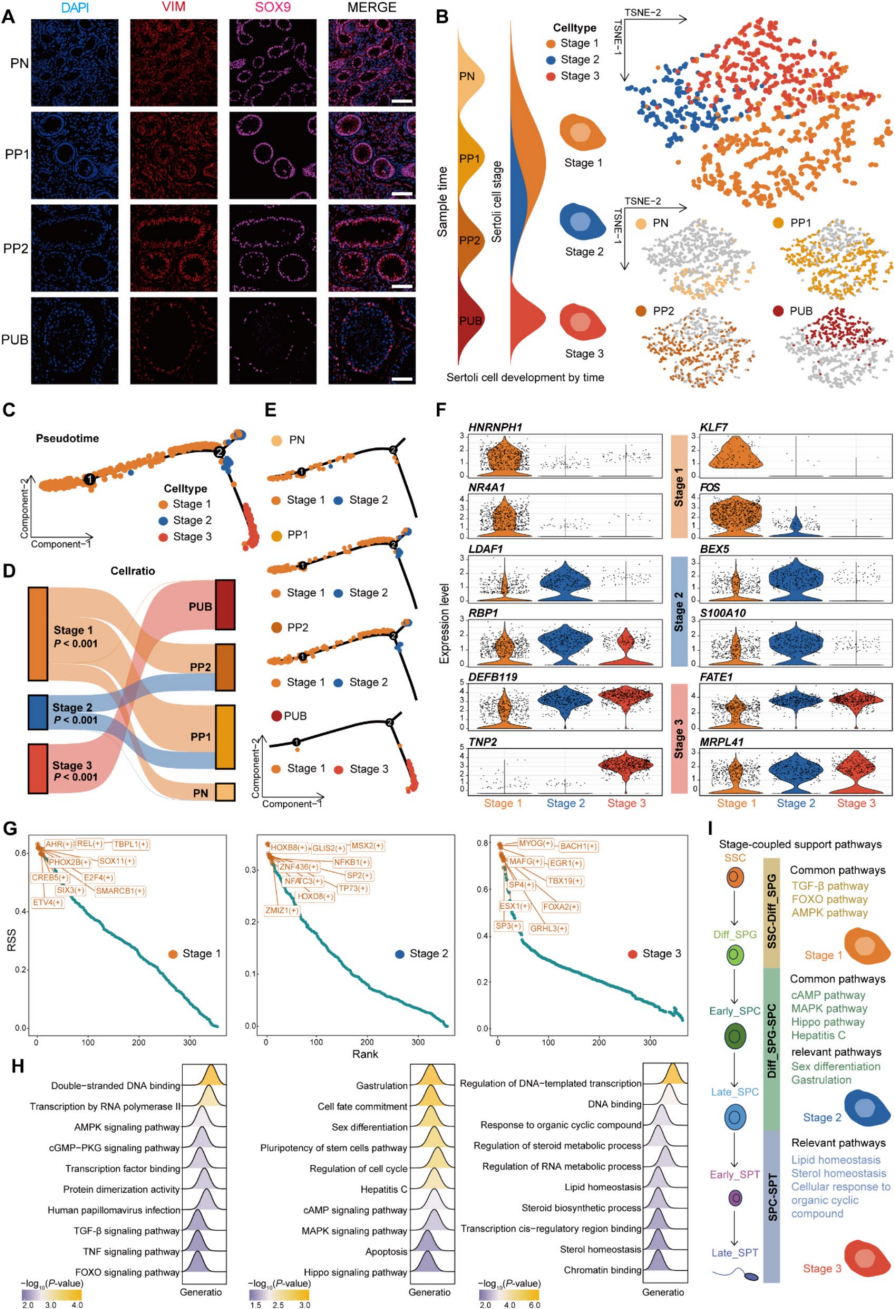
Characterization of three developmental stages during Sertoli cell ontogeny. **A** The immunolocalization of Sertoli cells was analyzed through co-staining of SOX9 and VIM. Scale bar: 50 μm. **B** TSNE visualization based on RNA levels illustrates the Sertoli cells of Simmental cattle across PN, PP1, PP2, and PUB developmental stages. **C **and** E** Pseudotemporal trajectory analysis of Sertoli cells (cell types annotated in Fig. C, developmental stages in Fig. E). **D** Sankey plot of Sertoli cell proportions across stages (Stage 1: Orange; Stage 2: Blue; Stage 3: Red). *P*-values were calculated using the Cochran-Armitage trend test across the four developmental time points (PN, PP1, PP2, and PUB). **F** Violin plot depicting the expression of selected markers across Sertoli cell stages. **G** Regulon specificity score (RSS) plot of regulon activity (top 10 scores highlighted in yellow). **H** Functional enrichment analysis across Sertoli cell stages based on activated regulon. **I** Stage‑specific pathways shared by Sertoli cell subtypes and germ cell developmental stages

Next, through immunofluorescence staining for Vimentin (VIM), which labels all somatic cells, and SOX9, a specific marker for Sertoli cells, we observed that Sertoli cells exhibit distinct organizational patterns across different developmental stages. The co-localization of VIM and SOX9 confirmed the identity of Sertoli cells. In prepubertal stages, Sertoli cells displayed a disorganized distribution intermixed with germ cells within the seminiferous cords. However, by the PUB stage, these cells transitioned to an ordered alignment along the basement membrane of the tubules (Fig. 4A). To systematically characterize the developmental progression of Sertoli cells, we performed subcluster analysis at both RNA (Fig. 4B and Table S7) and ATAC (Fig. S5A) levels, defining three consecutive maturation stages: "Stage 1–3". Pseudotime analysis integrating age-stratified transcriptional profiles revealed a sequential transition from Stage 1 to Stage 3 (Fig. 4C). Stage 1 predominated immediately after birth, Stage 2 emerged prior to sexual maturation, and Stage 3 became predominant during pubertal maturation (Fig. 4D and E). Integrated transcriptomic-epigenomic profiling revealed stage-specific molecular signatures: Stage 1 exhibited dual high expression of *HNRNPH1*, *KLF7*, *NR4A1*, and *FOS* at both the RNA-seq and ATAC-seq levels. Notably, existing studies have shown that conditional knockout of *HNRNPH1* in mouse Sertoli cells compromises blood-testis barrier function, delays meiotic progression, increases germ cell apoptosis, and leads to germ cell exfoliation, ultimately resulting in infertility, underscoring the functional importance of this gene in Sertoli cell maturation [42]. Similarly, Stage 2 was characterized by co-elevated expression of *LDAF1*, *BEX5*, *RBP1*, and *S100A10*, while Stage 3 uniquely demonstrated upregulated *DEFB119*, *FATE1*, *TNP2*, and *MRPL41* (Fig. 4F, Fig. S5B and Table S7). These stage-discriminative biomarkers provide critical molecular criteria for defining bovine Sertoli cell maturation phases.

Next, we further investigated the critical regulatory factors governing stage-specific cell fate transitions using SCENIC. Based on the regulon specificity score, which prioritizes regulons located closer to the top-left corner as highly stage-specific, we identified key determinants for each stage: Stage 1 included AHR, REL, TBPL1, PHOX2B, SOX11, CREB5, E2F4, SIX3, SMARCB1, and ETV4; Stage 2 was characterized by HOXB8, GLIS2, MSX2, ZNF436, NFKB1, NFATC3, TP73, HOXD8, and ZMIZ1; while Stage 3 regulation predominantly involved MYOG, BACH1, MAFG, EGR1, SP4, TBX19, ESX1, FOXA2, SP3, and GRHL3 (Fig. 4G and Table S7). These findings establish a transcriptional framework for Sertoli cell fate determination in Simmental cattle. Furthermore, we delineated stage-dependent signaling pathways orchestrating cellular transitions through top TFs that were obtained from SCENIC analysis. It is noteworthy that the FOXO, AMPK and TGF-β signaling pathways were identified in Stage 1, while the MAPK and Hippo signaling pathways were discovered in Stage 2 (Fig. 4H and Table S7). We found that FOXO, AMPK, and TGF-β signaling pathways were enriched in the transition from SSCs to Diff-SPG, and the MAPK and Hippo signaling pathways mediated the progression from Diff-SPG to SPC (Fig. 4I), these consistent signaling patterns suggest that Sertoli cells may interact with germ cells across various developmental stages.

### Analysis of the development process of Leydig cells and peritubular myoid cells

Initial clustering analysis revealed that testicular Leydig cells and peritubular myoid cells were co-localized within the same major cluster. To investigate the developmental status of Leydig cells (CYP11A1^+^) and peritubular myoid cells (CD34^+^), we initially identified these two cell types through staining (Fig. S5C) and then distinguished them further using known marker genes for subclustering at the RNA level (Fig. S5D, S5E and Table S7). The genes *CYP17A1*, *DLK1*, *STAR*, *CFD*, *DCN*, *IGF2*, *INHBA*, and *HSD3B1* exhibit specific expression in Leydig cells, whereas *COL3A1*, *COL1A1*, *TPM1*, *ACTA2*, *MYH11*, *CD34*, *THY1*, and *CYP26B1* are predominantly localized to peritubular myoid cells (Fig. S5E). Subsequently, to resolve the functional and developmental heterogeneity within Leydig cells, we performed reclustering based on their RNA expression profiles. This approach delineated two distinct populations: fetal Leydig cells, marked by expression of *INSL3* and *RXFP2*, and adult Leydig cells, characterized by *HSD17B3* and *CYP11B1* expression [43] (Fig. S5F and S5G). By comparing the proportions of adult Leydig cells and fetal Leydig cells in different samples, we found that in this study, the expression of fetal Leydig cells gradually decreased from PN to PP, while the number of adult Leydig cells was the highest at PP and then gradually decreased (Fig. S5H). Additionally, we noticed that the distributions of adult Leydig cells and peritubular myoid cells were highly overlapping. To validate this observation, we further investigated the functions of these three cell types. The results highlighted shared functional features between adult Leydig cells and peritubular myoid cells, including pathways such as "Cytoskeleton in muscle cells", "Relaxin signaling pathway", "Oxytocin signaling pathway", and "PI3K-Akt signaling pathway" (Fig. S5I and Table S7). Building upon previous work proposing a common progenitor for Leydig cells and peritubular myoid cells [26], our study specifically demonstrates that this developmental relationship is most evident in adult Leydig cells.

To elucidate the transition from fetal to adult Leydig cells, we analyzed differentially expressed genes across three developmental stages. Functional enrichment analysis showed that genes upregulated in postnatal-to-prepubertal fetal Leydig cells were significantly enriched in processes like "Response to hormone", "Regulation of hormone levels", and the "GnRH signaling pathway". Notably, these same pathways were also enriched among genes downregulated during the prepubertal-to-pubertal transition (Fig. S5J). This biphasic pattern reveals a clear functional trajectory: fetal Leydig cells persisting after birth retain foundational hormonal response and regulatory capacities, which subsequently diminish or are remodeled as development proceeds. Collectively, these findings deepen our understanding of the regulatory mechanisms governing testicular Leydig and peritubular myoid cell development, and directly demonstrate, at the molecular level, the dynamic functional remodeling of the postnatal Leydig cell population.

### Dynamic intercellular communication between somatic and germ cells in the testis

**Fig. 5:**
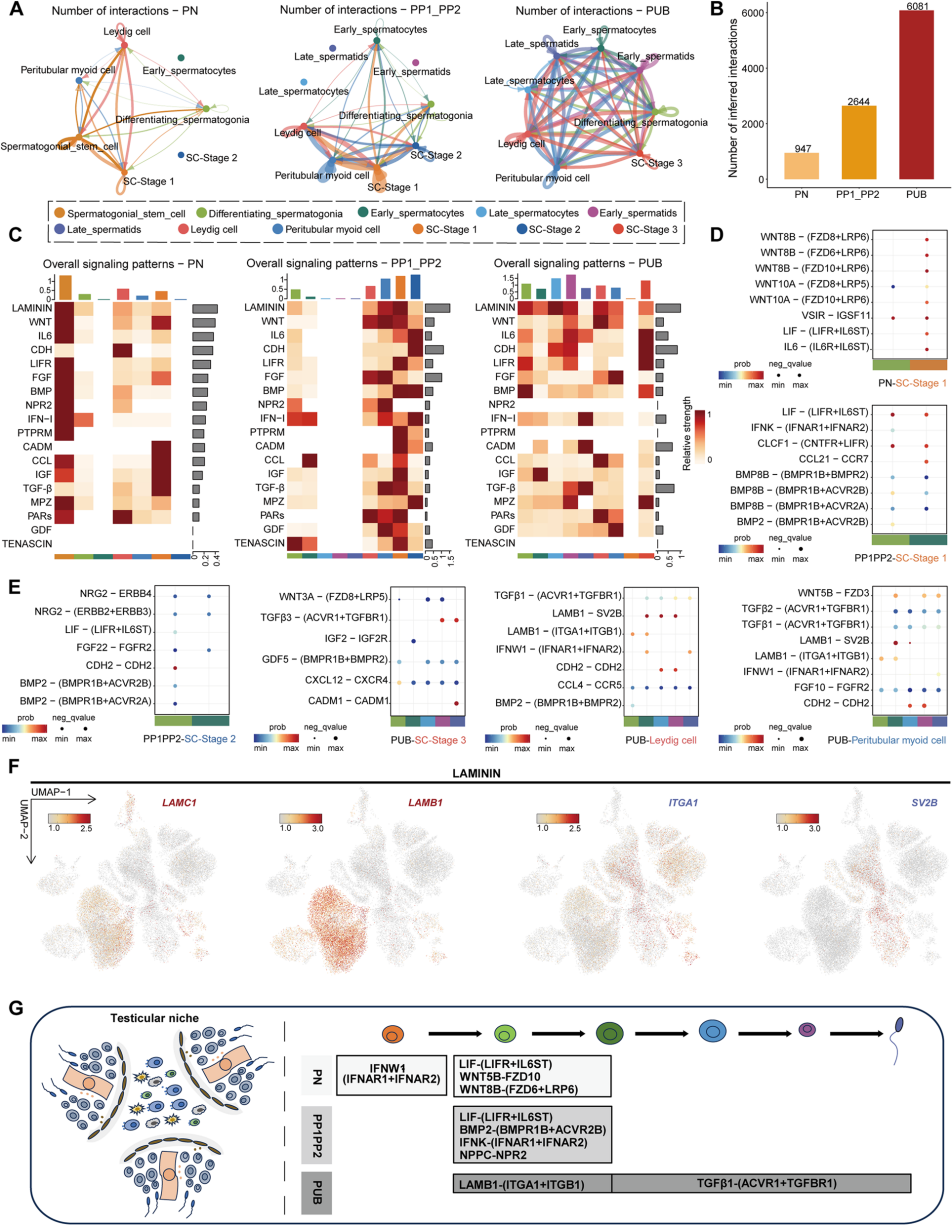
Cell–cell communication network between testicular somatic cells and germ cells in Simmental cattle. **A **and** B** Number of interactions among somatic cells and germ cells. **C** Comparison of overall signaling in testicular cells across PN, PP1-PP2, and PUB stages via heatmap: the top bar plot represents the total signaling activity of each cell cluster, while the right bar plot indicates the prevalence of each pathway across cell types. The color intensity reflects the signaling activity level of each pathway within specific clusters. **D **and** E** Dot plot showing ligand-receptor communications between germ cells (target) and somatic cells (source) at different age. The color of bubbles represents the q-value of the communication probability. The text at the bottom of the image represents the interaction between specific types of somatic cells and germ cells during developmental stages. **F** Expression of ligands (red) and receptors (blue) of indicated signaling pathways cast on the UMAP plot from Fig. 1B. **G** Schematic model of key ligand-receptor pairs involved in somatic-germ cell interactions across various developmental stages in Simmental cattle testes

After characterizing the molecular mechanisms governing the development of germ cells and somatic cells separately, and informed by the coordinated development of germ cells and Sertoli cells, we employed CellChat to systematically evaluate intercellular signaling patterns based on single-cell transcriptomic data, including both the number of interactions and the weight of interactions (Fig. 5A and Fig. S6A). As age increases, both the number and intensity of cell-to-cell interactions increase significantly, indicating that cell communication and collaboration become increasingly complex and important during development (Fig. 5B and Fig. S6B). Further analysis of the significance of signaling pathways between germ cells and somatic cells across various developmental stages revealed the following insights: During the PN stage, Sertoli cells in Stage 1 interact with SSCs through signaling pathways such as WNT, CCL, IGF, and TGF-β. As development progresses into the PP period, Leydig cells and peritubular myoid cells interact with Diff-SPG through the NPR2 signaling pathway. By the PUB stage, Leydig cells and peritubular myoid cells continue to be involved in the differentiation of SSCs into sperm via CCL and IFN-1 signaling pathways, while Sertoli cells in Stage 3 are mainly involved in the spermatogenesis process of SSCs through IL6 and LIFR pathways (Fig. 5C and Fig. S6C). We noticed that genes encoding ligands and receptors that participate in multiple signaling pathways (Fig. 5D, E and Fig. S6D), including LAMININ, were expressed in a cell type-specific manner (Fig. 5F). In summary, we outlined the interactions between cell types in the testes of Simmental cattle, and further delineated the ligand-receptor pairs that play significant roles at various developmental stages (Fig. 5G). These predictions provide a foundation for future functional studies to validate the precise roles of these signaling events.

### Cross-species comparison reveals core transcription factors in the germ cell developmental lineage

**Fig. 6:**
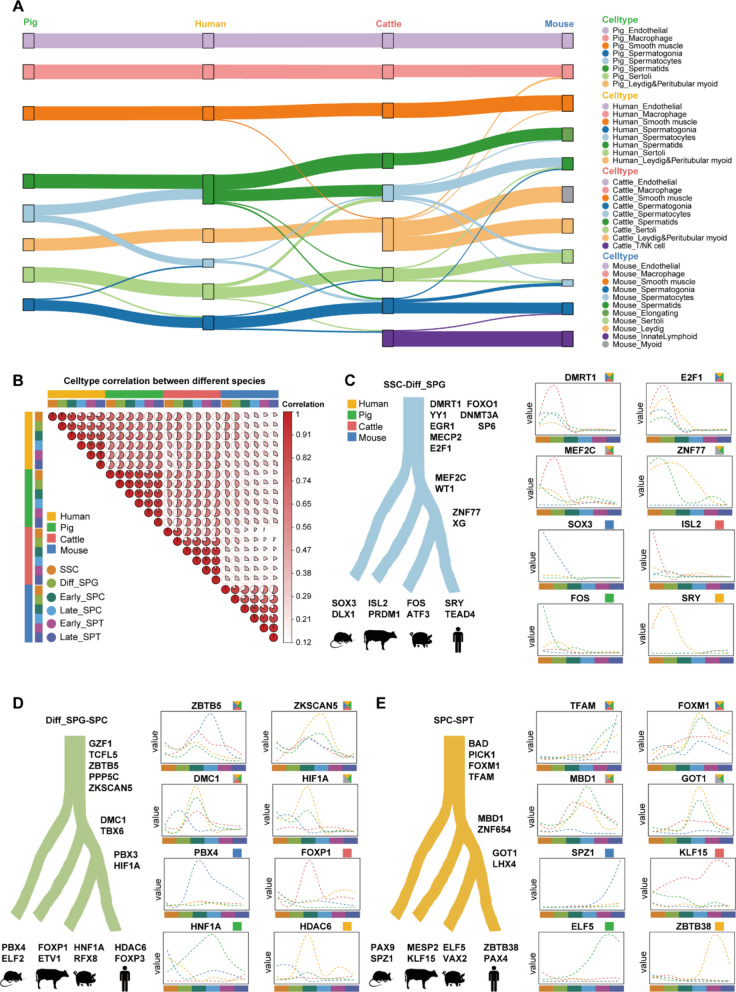
Core transcription factors in testicular germ cell development of human, pig, cattle, and mouse. **A** Sankey diagram illustrating the evolutionary conservation and species specificity of testicular cell development across different species. **B** Heatmap illustrating the correlation of germ cell types across different species. **C** Diagram of the expression patterns of the master regulators in the SSCs and Diff-SPG of human, pig, cattle, and mouse. **D** Diagram of the expression patterns of the master regulators in the Diff-SPG and SPC of human, pig, cattle, and mouse. **E** Diagram of the expression patterns of the master regulators in the SPC and SPT of human, pig, cattle, and mouse

To comprehensively explore the conservation and divergence of testicular development across species, we analyzed single-cell transcriptomic data from testicular tissues of human [44], pig [24, 45], cattle, and mouse [21]. Based on marker gene expression profiling, human, pig, and mouse testicular cells were classified into 8, 8, and 11 distinct cell types, respectively, with germ cells further subdivided into 6 subpopulations (Fig. S7A–S7F and Table S8). To evaluate the conservation and divergence of testicular cell development across species, we performed pairwise SAMap analysis. This approach revealed broadly conserved cellular identities, with strong evolutionary conservation observed in endothelial cells, a finding consistent with previous studies. Additionally, macrophages and smooth muscle cells also exhibited high transcriptional similarity, further underscoring the deep conservation of key stromal and immune components in testis development (Fig. 6A). Heatmap visualization revealed evolutionary conservation in testicular cell types across species, with pronounced transcriptional similarities between human and pig, and likewise between cattle and pig (Fig. 6B). Pseudotime trajectory analysis of germ cell development provided additional evidence for a conserved developmental program between cattle and pig (Fig. S8A).

**Fig. 7:**
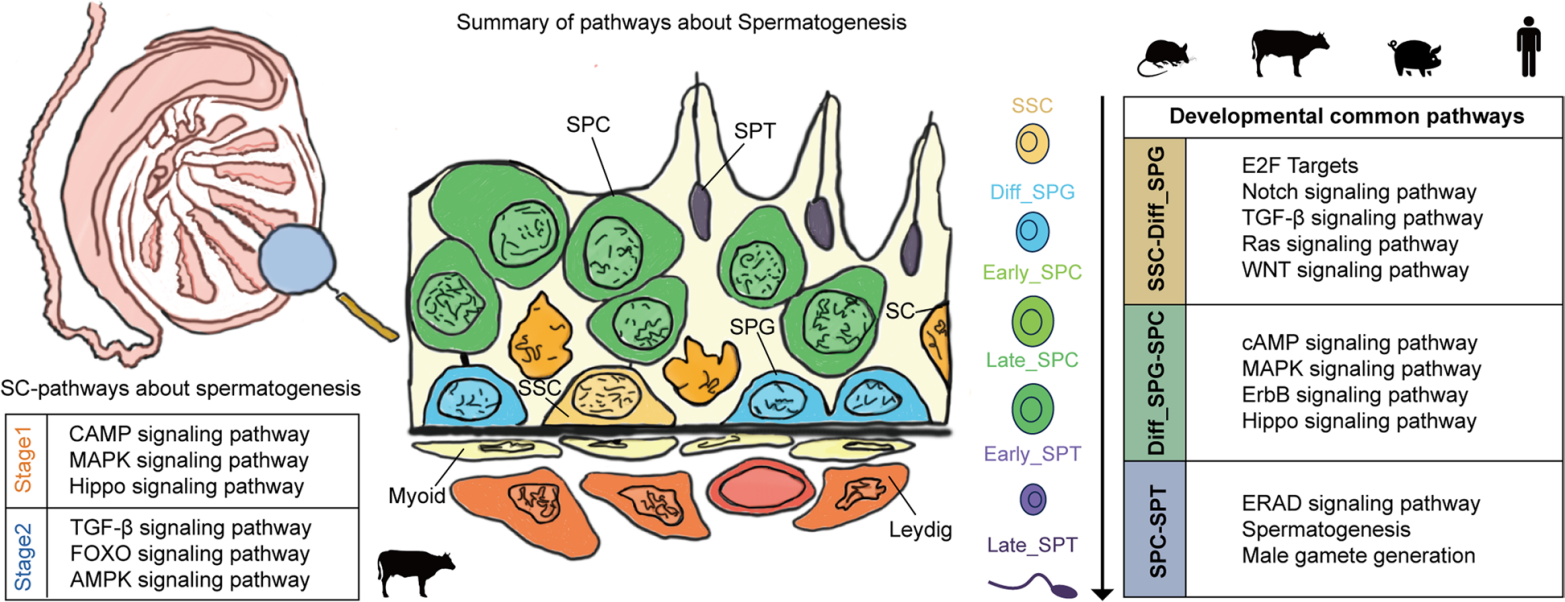
Comparative schematic model of signaling pathways governing germline stem cell-niche interactions across Simmental cattle, pig, mouse, and human

To investigate the molecular regulation of germ cell differentiation and the genetic foundations of developmental pathways among species, we consolidated pseudotime trajectory genes obtained from comparative germ cell analyses involving human versus cattle, pig versus cattle, and mouse versus cattle. This process detected 929 genes uniquely associated with human and cattle, 1,468 unique to pig and cattle, and 729 specific to mouse and cattle, in addition to 6,333 genes common to all species (Fig. S8B). Following this, Mfuzz clustering applied to the conserved genes uncovered 50 separate co-expression modules during germ cell development (Fig. S8C and Table S10). Enrichment analysis performed over successive germ cell developmental stages showed that "E2F targets" and the "TGF-β signaling pathway" were consistently enriched during the SSC–SPG transition in all four species. Enrichment of the "ErbB signaling pathway" was observed in the subsequent SPG–SPC stage, and terms associated with "Spermatogenesis" were significantly enriched during the SPC–SPT stage (Fig. S8D and Table S10). We also pinpointed transcription factors that were either conserved or species-specific at distinct germ cell developmental stages in human, pig, cattle, and mouse (Fig. 6C–E and Table S9). These results offer new perspectives on the regulatory circuitry guiding germ cell differentiation in an evolutionary context. In conclusion, this study provides a roadmap of the signaling pathways between germ cells and Sertoli cells in Simmental cattle, and also identifies conserved germ cell functions shared with mouse, pig, and human, paving the way for future functional studies (Fig. 7).

## Discussion

Spermatogenesis represents an exquisitely complex biological process, fundamentally orchestrated through the coordinated interplay of multiple testicular cell populations that establish and maintain the specialized testicular microenvironment [5, 15]. While substantial progress has been made in elucidating spermatogenic mechanisms through contemporary investigations [46–50], significant knowledge gaps persist regarding this process in economically crucial beef breeds, particularly Simmental cattle that dominates global meat production systems. To address this critical void in comparative reproductive biology, we have constructed the first comprehensive single-cell transcriptomic atlas characterizing testicular development in prepubertal Simmental bulls, providing an essential resource for understanding species-specific regulation of spermatogenesis.

Regarding the research on germ cells, previous studies suggest SPG proliferation in bulls initiates 4–5 weeks postpartum, with a massive expansion by week 24, the appearance of primary SPC around week 20, secondary SPC between weeks 20–30, and mature sperm production between weeks 32–44 [51]. Our results confirm that SPG proliferation predominantly occurs at the prepubertal stage. In addition, we observed that SPC in Simmental cattle already exist after birth, but do not begin meiosis until prepubertal, forming Late SPC, and this time is consistent with the period reported in the aforementioned studies. The above results suggest that the preparation period of SPC in Simmental cattle before entering meiosis is relatively long. If this preparation time could be shortened, it might help to accelerate the breeding process. Moreover, we detected some SPT in the prepubertal samples of Simmental cattle, and it is speculated that Simmental cattle may enter puberty earlier than previously documented. These findings provide certain insights for the breeding work of Simmental cattle.

This study also systematically investigated the crucial roles of somatic cells, particularly Sertoli and Leydig cells, in the spermatogenic microenvironment. Sertoli cells are an important scaffold for spermatogenesis [52, 53]. Previous studies have shown that Sertoli cells mainly develop from an immature to a mature state before puberty and are fully developed at puberty. However, our study refines this process by dividing their development into three distinct stages (Stage 1–3). Notably, previous studies have also indicated that human Sertoli cells can be divided into three stages [44]. Inspired by this, we conducted an in-depth analysis of Sertoli cells from 24-month-old water buffalo based on Huang's article [38], and found that adult bovine Sertoli cells can similarly be classified into these three stages. These results significantly advance the understanding of Sertoli cell development.

Functional enrichment analysis revealed that signaling pathways including TGF-β, AMPK, and MAPK are specifically activated at different Sertoli cell stages and show precise coordination with germ cell differentiation: AMPK and TGF-β pathway activation in Stage 1 Sertoli cells corresponds to the SSC-to-Diff-SPG transition, while MAPK activation in Stage 2 aligns with the Diff-SPG-to-SPC differentiation. These pathways show conserved enrichment across multiple species, emphasizing their central importance in germ cell development. Previous studies have indicated that the TGF-β/Smad pathway regulates intercellular junction dynamics, AMPK is involved in blood-testis barrier maintenance and lactate metabolism, and MAPK, particularly ERK and p38, regulates germ cell self-renewal, meiosis, and Sertoli cell proliferation [14, 54]. Collectively, these findings suggest that Sertoli cells may actively participate in regulating germ cell development through stage-specific signaling pathways rather than merely providing passive structural and nutritional support. Additionally, through cross-species comparative analysis, we found that signaling pathways such as TGF-β and MAPK are evolutionarily conserved across multiple species during spermatogenesis, further supporting their fundamental regulatory roles in germ cell development.

Our cross-species comparison of transcription factor dynamics across four mammals (human, pig, cattle, mouse) reveals a core conserved framework alongside species-specific adaptations. At the SSC-to-Diff-SPG transition, factors like DMRT1, FOXO1, and E2F1 showed conserved downregulation, highlighting their core role in stemness exit. Conversely, factors like SOX3 exhibited divergent patterns, indicating alternative regulatory strategies. During meiosis initiation (Diff-SPG-to-SPC), key factors such as DMC1 were uniformly upregulated, emphasizing deep conservation, while others like PBX4 showed species variance. At the spermiogenic transition (SPC-to-SPT), mitochondrial regulators like TFAM displayed stable expression, reflecting conserved metabolic remodeling needs. In contrast, terminal differentiation factors like SPZ1 showed significant trajectory divergence, suggesting adaptive evolution in the final steps of sperm formation to meet species-specific reproductive demands.

Leydig cells, as the key cell type in the testicular microenvironment responsible for hormone secretion, can be divided into fetal and adult Leydig cells [15]. The issue of whether fetal Leydig cells persist after birth has long been a matter of debate [43, 55–57]. To clarify this biological event, we conducted a detailed classification of the interstitial cells in Simmental cattle and reached the following conclusions: fetal Leydig cells do indeed persist after birth, but their numbers gradually decrease with age. This study provides new insights into the development of Leydig cells and peritubular myoid cells. Cell–cell interaction analysis further predicted the involvement of Leydig cells in germ cell development and their cooperative mechanisms with Sertoli cells.

## Conclusions

This study employed an integrated approach of scRNA-seq and sNucATAC-seq to elucidate the regulatory network governing germ cell fate during testicular development in Simmental cattle. Spermatogenesis was revealed to be a multi-layered and coordinated process, regulated not only by germ cell-intrinsic transcription factors such as E2F1 and FOXO1, but also by stage-specific signaling pathways mediated by somatic cells, specifically TGF-β, AMPK, and MAPK from Sertoli and Leydig cells. A key finding is the first identification in cattle of three functional Sertoli cell subtypes that provide sequential support for germ cell differentiation. Furthermore, the persistence and subsequent gradual decline of fetal Leydig cells in the postnatal period were confirmed. Evolutionary conservation of these regulatory mechanisms was highlighted through cross-species comparison. These results provide a new theoretical foundation for advancing the understanding of bovine spermatogenesis at the cellular and molecular levels and for strategies aimed at improving reproductive efficiency.

## Supplementary Information

Additional file 1: Fig. S1. Histological analysis of Simmental cattle testis. Fig. S2. scRNA-seq and sNucATAC-seq of developing Simmental cattle testis. Fig. S3. Core genes and signaling pathways orchestrating stage-specific spermatogenesis in Simmental cattle. Fig. S4. Core transcription factors and pathways in germ cell fate transition of Simmental cattle. Fig. S5. Characterization and developmental lineage tracing of Sertoli cells, Leydig cells and peritubular myoid cells across three developmental stages. Fig. S6. Cell-cell communication network between testicular somatic cells and germ cells in Simmental cattle. Fig. S7. Distribution and gene expression patterns of testicular cell types in humans, pigs, and mice. Fig. S8. Comparative analysis of gene expression dynamics across humans, cattle, pigs, and mice during germ cell differentiation.

Additional file 2: Table S1. Information collection of Simmental cattle. Table S2. Simmental cattle information summary sheet.

Additional file 3: Table S3. Markers and cell ratios of testicular cell types in ATAC-seq and RNA-seq analyses.

Additional file 4: Table S4. Analysis of germ cell markers, biological functions and key transcription factors based on RNA-seq data.

Additional file 5: Table S5. Genes and functions associated with transitions among the six germ cell types.

Additional file 6: Table S6. Functional analysis of differentially expressed genes in six types of germ cells.

Additional file 7: Table S7. Markers and functional characterization of somatic cells in Simmental cattle testes.

Additional file 8: Table S8. Conserved and species-specific cellular markers in human, pig, and mouse.

Additional file 9: Table S9. Expression of conserved transcription factors in germ cells across four species (human, pig, mouse, cattle).

Additional file 10: Table S10. Expression patterns and functional implications of genes along conserved developmental trajectories across species.

## Data Availability

All sequencing data were deposited at the National Genomics Data Center under accession number OMIX009660. Human: GSE149512, Pig: GSE186479 and GSE174782, Mouse: GSE112393. Any additional information required to reanalyze the data reported in this paper is available from the lead contact Yulei Wei upon request.

## Abbreviations

Diff-SPG: Differentiating spermatogonia
PN: Postnatal
PP: Prepubertal
PUB: Pubertal
QC: Quality-control
scRNA-seq: Single-cell RNA sequencing
sNucATAC-seq: Single-nucleus ATAC sequencing
SPG: Spermatogonia
SPC: Spermatocytes
SPT: Spermatids
SSCs: Spermatogonial stem cells
