# Supplementary material for: Depicting the dynamic transcriptional and epigenetic landscape of testis development in pubertal Simmental cattle
Source: J Anim Sci Biotechnol. 2026 May 18;17:96. doi: 10.1186/s40104-026-01406-x (PMC13181952; doi:10.1186/s40104-026-01406-x)
Supplement: Supplementary file 1 — Additional file 1: Fig. S1. Histological analysis of Simmental cattle testis. Fig. S2. scRNA-seq and sNucATAC-seq of developing Simmental cattle testis. Fig. S3. Core genes and signaling pathways orchestrating stage-specific spermatogenesis in Simmental cattle. Fig. S4. Core transcription factors and pathways in germ cell fate transition of Simmental cattle. Fig. S5. Characterization and developmental lineage tracing of Sertoli cells, Leydig cells and peritubular myoid cells across three developmental stages. Fig. S6. Cell-cell communication network between testicular somatic cells and germ cells in Simmental cattle. Fig. S7. Distribution and gene expression patterns of testicular cell types in humans, pigs, and mice. Fig. S8. Comparative analysis of gene expression dynamics across humans, cattle, pigs, and mice during germ cell differentiation. [file 40104_2026_1406_MOESM1_ESM.docx]

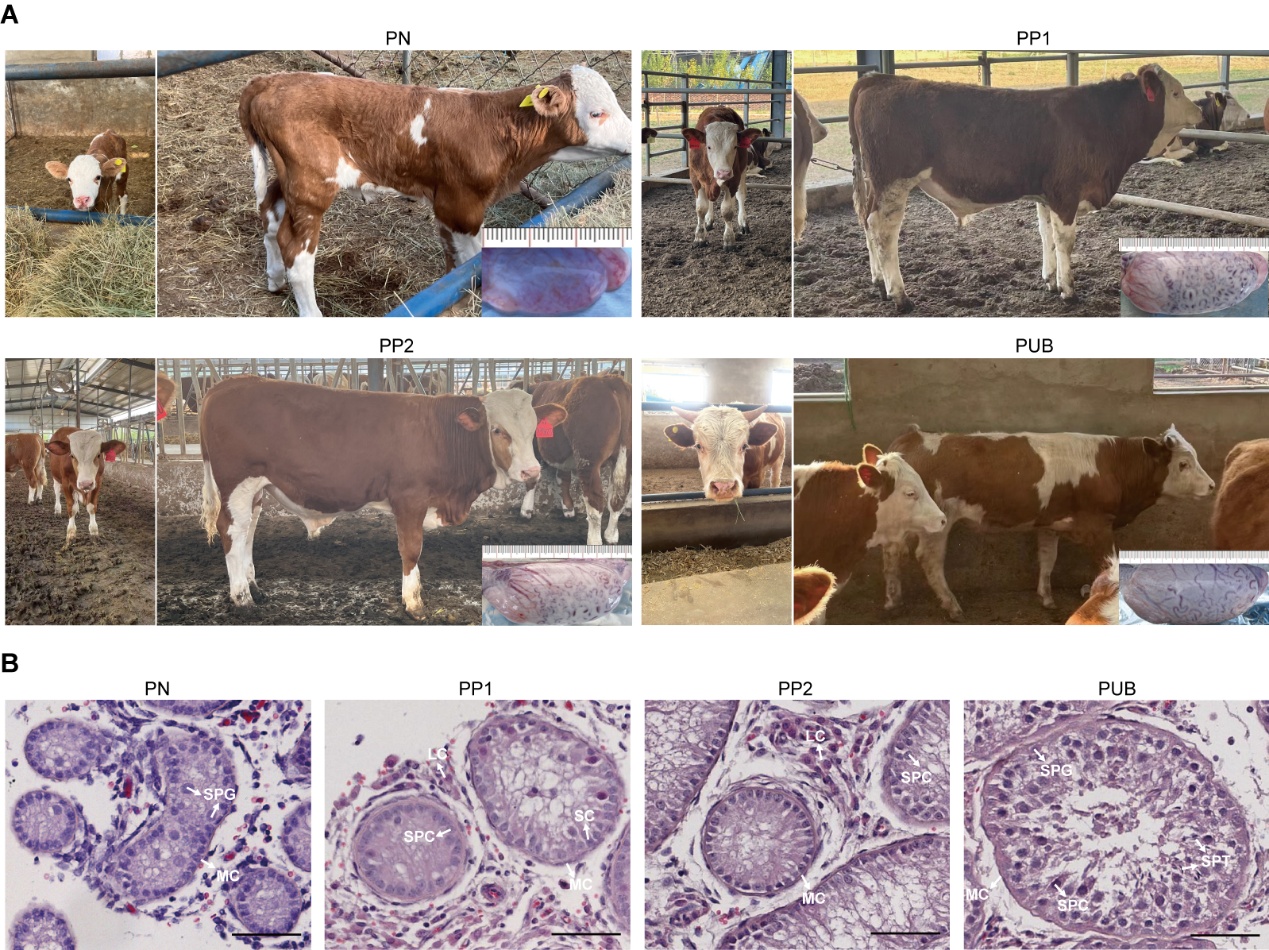


**Fig. S1** Histological analysis of Simmental cattle testis. **A** Testicular tissues from three developmental stages were collected for scRNA-seq and histological validation: PN (postnatal, 4 d, *n* = 1), PP1 (early prepubertal, 5 months, n=1), PP2 (late prepubertal, 7 months, *n* = 1), and PUB (puberty, 14 months, *n* = 1). **B** Hematoxylin and eosin (H&E) staining of testicular sections from Simmental cattle. Key cell types are identifiable, including spermatogonia (SPG), spermatocytes (SPC), spermatids (SPT), Sertoli cells (SC), Leydig cells (LC), and Peritubular myoid cells (MC). Scale bar: 50 μm


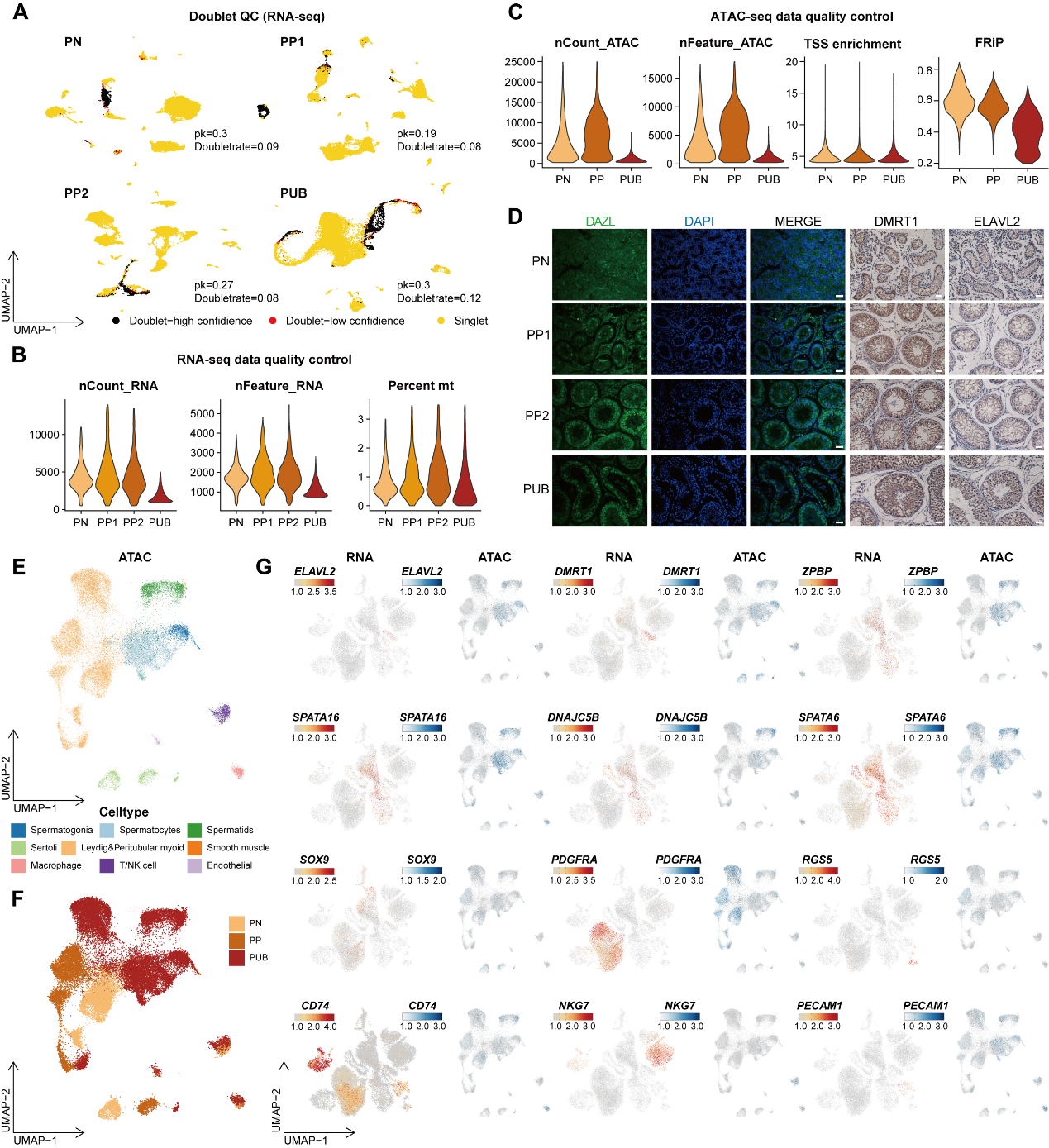


**Fig. S2** scRNA-seq and sNucATAC-seq of developing Simmental cattle testis. **A** UMAP projection showing doublet detection results from the scRNA-seq dataset. Cells are colored by classification: yellow (singlets), red (low-confidence doublets), and black (high-confidence doublets). Both low- and high-confidence doublets (red and black) were removed from downstream analysis. **B** Violin plots displaying key quality control metrics—nCount_RNA, nFeature_RNA, and percentage of mitochondrial reads (from left to right, respectively)—across all samples in the scRNA-seq dataset after doublet removal. **C** Violin plots displaying key quality control metrics—nCount_ATAC, nFeature_ATAC, TSS enrichmrent score, and Fraction of reads in peaks (FRiP) (from left to right, respectively)—across all samples in the scATAC-seq dataset. **D** Germ cell markers in bovine testicular tissues at different developmental stages. DAZL, DMRT1, and ELAVL2 are all markers of SPG. Scale bar: 50 μm. **E** UMAP visualization of testicular cell distribution based on sNucATAC‑seq data. The plot displays the distribution of various testicular cell types across three developmental stages of Simmental cattle testes. Cells are colored by type, allowing visual comparison of cellular composition among stages. **F** Developmental stage-specific distribution of testicular cells in UMAP space. Cells are colored by developmental stage: yellow for PN (postnatal day 5), brown for PP (pre-pubertal, 5 months), and red for PUB (pubertal, 13 months), illustrating distinct clustering patterns across stages. **G** UMAP visualization of cell-type-specific markers in testicular cells at RNA and ATAC levels. Color intensity reflects marker signal strength, visually demonstrating the regulatory concordance between gene expression and chromatin openness within the same cell type


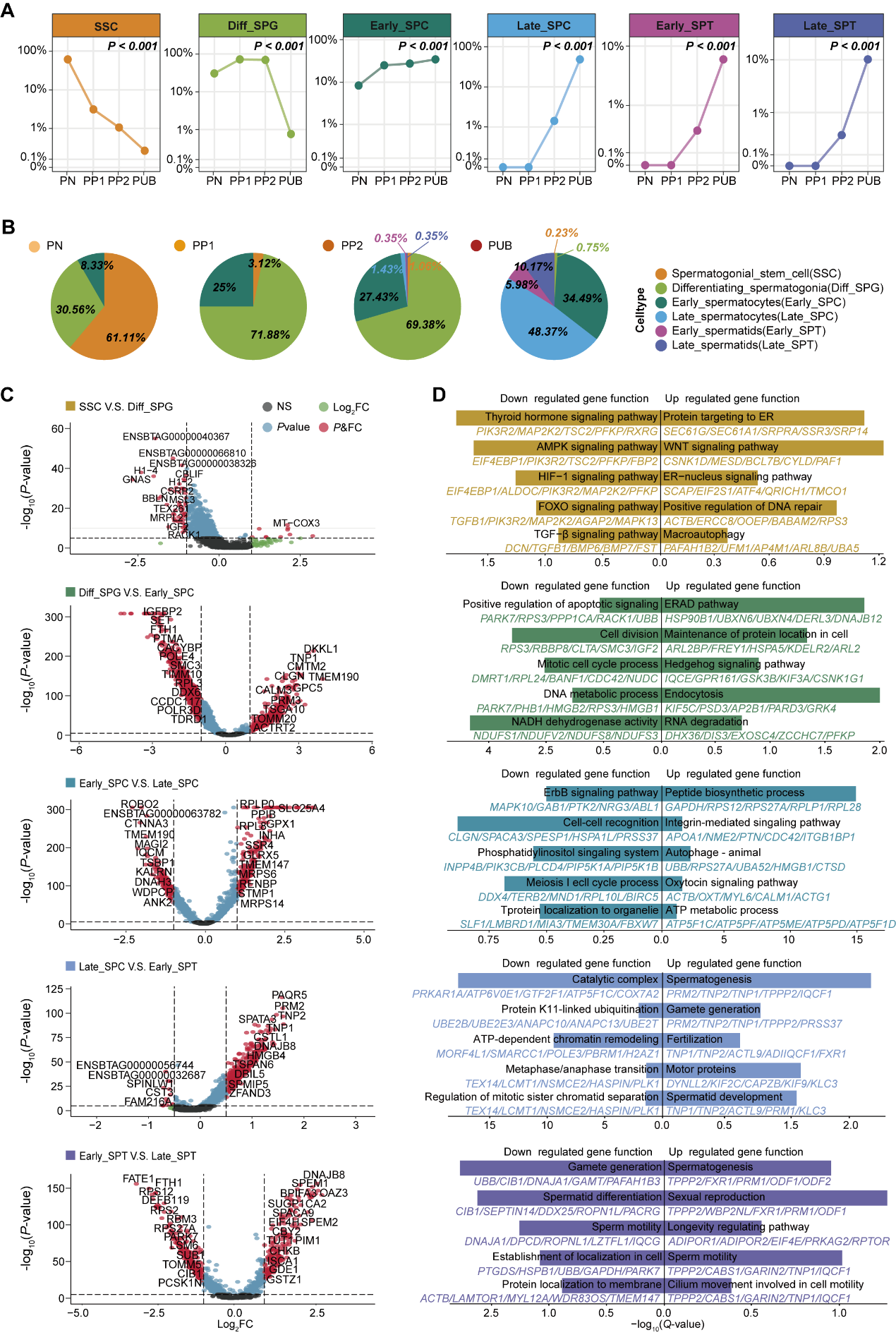


**Fig. S3** Core genes and signaling pathways orchestrating stage-specific spermatogenesis in Simmental cattle. **A** Changes in germ cell subpopulation proportions across developmental stages. *P*-values were calculated using the Cochran-Armitage trend test across the four developmental time points (PN, PP1, PP2, and PUB). **B** Cell proportions of germ cells across developmental stages. **C** Volcano plot visualizing significantly upregulated or downregulated genes during germ cell fate transition. **D** Enrichment of core signaling pathways based on upregulated and downregulated genes in germ cell fate transition of Simmental cattle


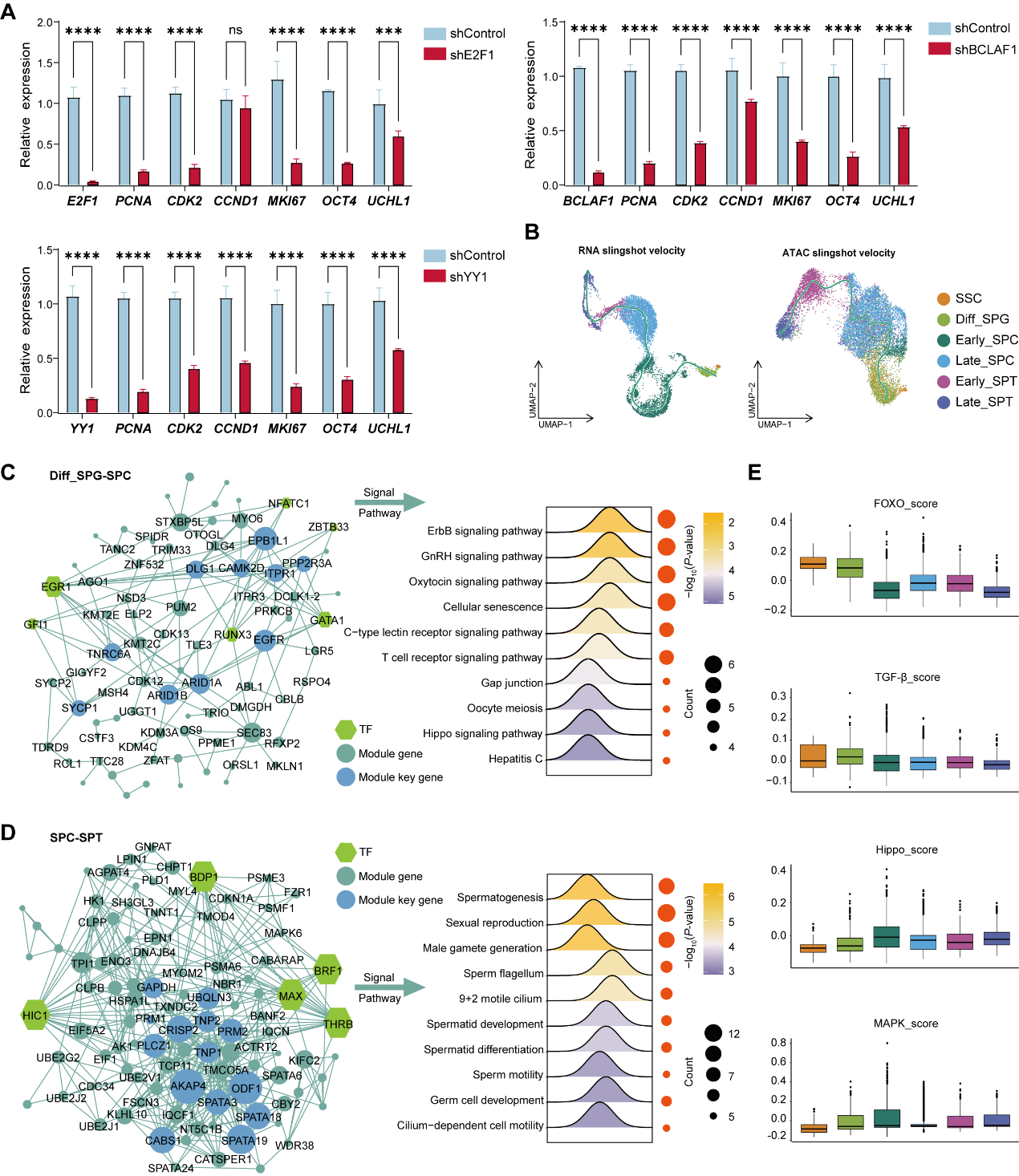


**Fig. S4** Core transcription factors and pathways in germ cell fate transition of Simmental cattle. **A** Relative expression levels of cell cycle and pluripotency-associated genes under different knockdown conditions. **B** Pseudotime analysis of germ cells using slingshot. The left plot shows the RNA trajectory, and the right plot shows the ATAC trajectory. **C** Protein-protein interaction network and active signaling pathways during the Diff-SPG-SPC developmental stage (Hexagon: TF; Green circle: module genes of network; Blue circle: module’s key genes of network). **D** Protein-protein interaction network and active signaling pathways during the SPC-SPT developmental stage (Hexagon: TF; Green circle: module genes of network; Blue circle: module’s key genes of network). **E** Box plot illustrating the distribution of key signaling pathways across various germ cell types in Simmental cattle


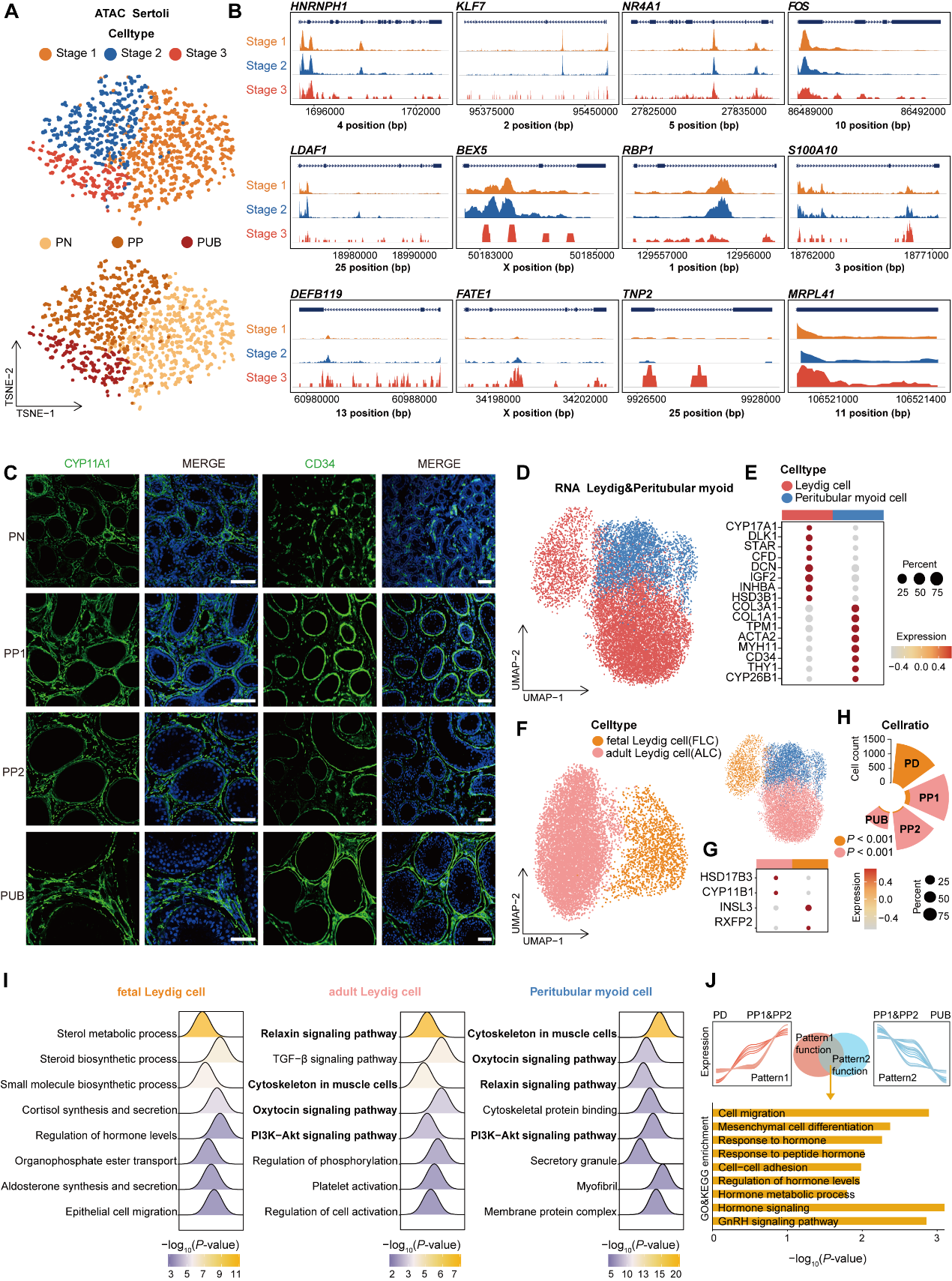


**Fig. S5** Characterization and developmental lineage tracing of Sertoli cells, Leydig cells and Peritubular myoid cells across three developmental stages. **A** TSNE visualization based on ATAC levels shows the Sertoli cells of Simmental cattle across PN, PP and PUB developmental stages. **B** Open chromatin regions of selected genes. **C** Immunolocalization of Leydig cells (CYP11A1) and Peritubular myoid cells (CD34). Scale bar: 25 μm. **D** UMAP visualization based on RNA levels illustrates the distribution of Leydig cells and Peritubular myoid cells in Simmental cattle across various developmental stages. **E** Dot plot depicting selected differentially expressed genes for Leydig cells and Peritubular myoid cells. **F** UMAP visualization based on RNA levels illustrates the adult Leydig cells and fetal Leydig cells of Simmental cattle across various developmental stages. **G** Dot plot of distinguishing markers between adult and fetal Leydig cells. **H** Pie chart illustrates the relative proportions of adult and fetal Leydig cells at different developmental stages. *P*-values were calculated using the Cochran-Armitage trend test across the four developmental time points (PN, PP1, PP2, and PUB). **I** Functional enrichment analysis of adult Leydig cells, fetal Leydig cells and Peritubular myoid cells. **J** Functional intersection of pattern 1 (upregulated from PN to PP1&PP2) and pattern 2 (downregulated from PP1&PP2 to PUB)


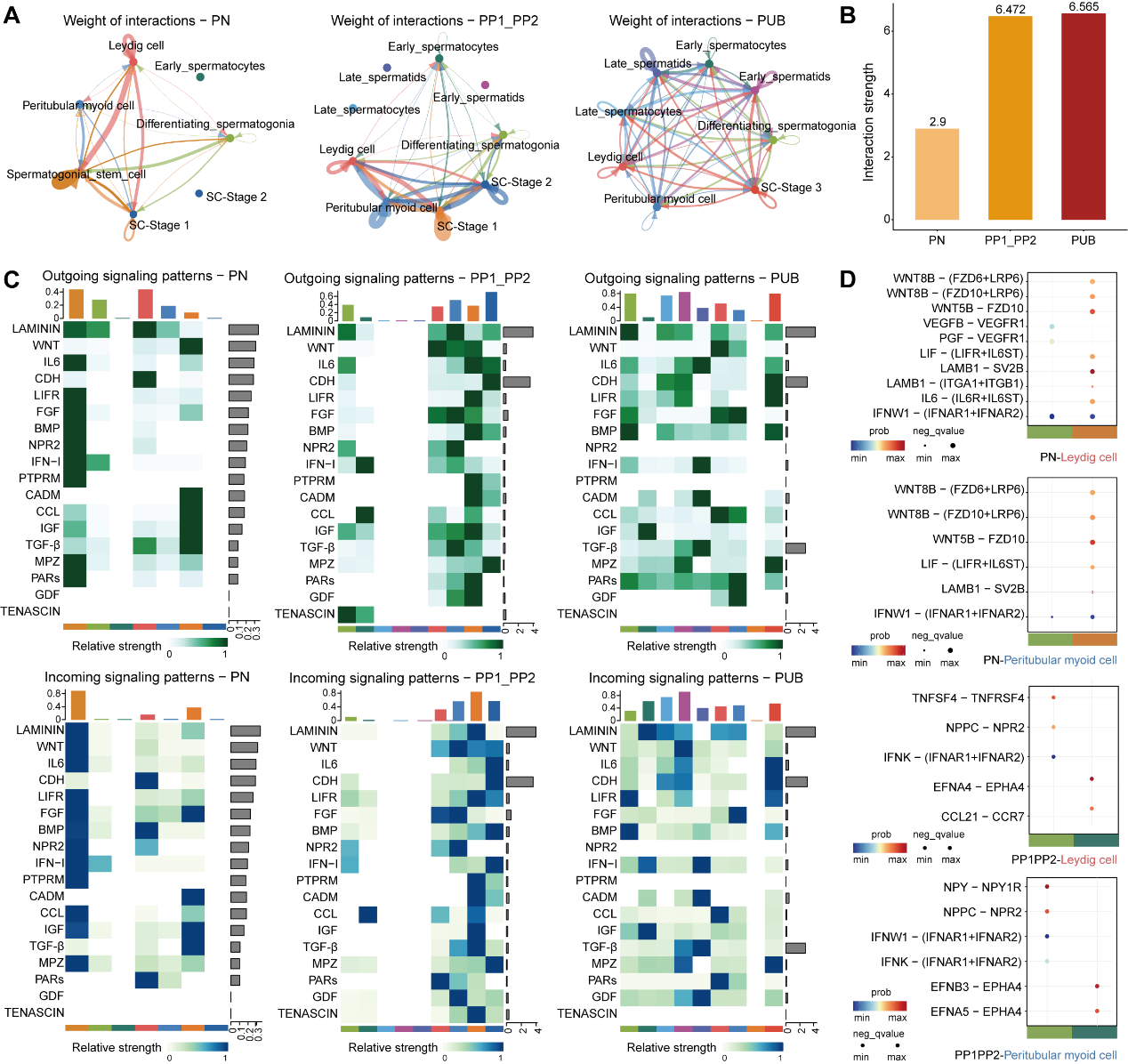


**Fig. S6** Cell-cell communication network between testicular somatic cells and germ cells in Simmental cattle. **A** and **B** Weight of interactions among somatic cells and germ cells. **C** Comparison of overall signaling in testicular cells across PN, PP1-PP2, and PUB stages via heatmap: the top bar plot represents the total signaling activity of each cell cluster, while the right bar plot indicates the prevalence of each pathway across cell types. The color intensity reflects the signaling activity level of each pathway within specific clusters. **D** Dot plot depicting ligand–receptor interactions between somatic cells (source) and germ cells (target) across developmental stages. Bubble color indicates the statistical significance (q-value) of communication probability. Labels indicate specific somatic–germ cell interactions during development


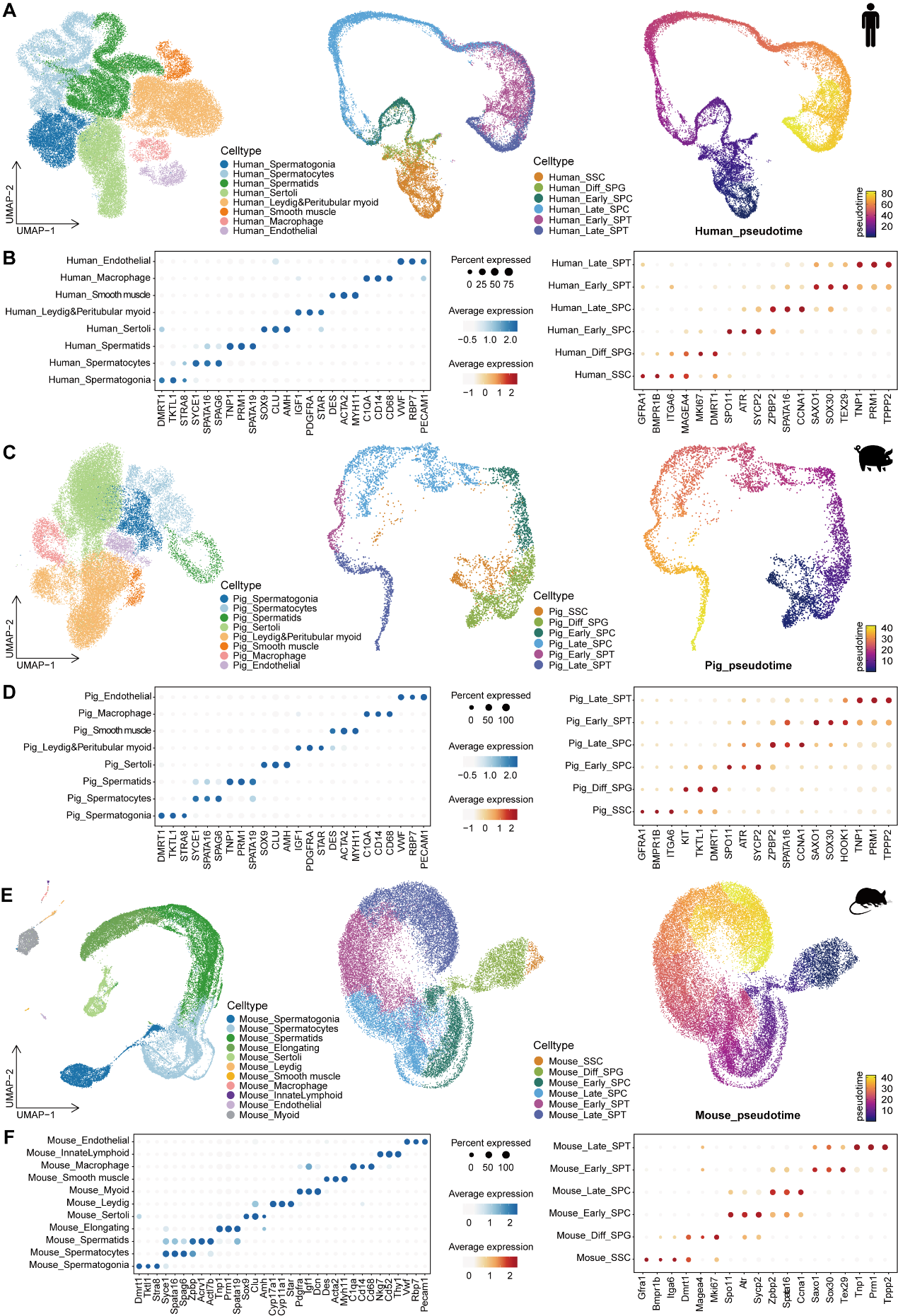


**Fig. S7** Distribution and gene expression patterns of testicular cell types in humans, pigs, and mice. **A** UMAP visualization showing the distribution of all cell types, major cell populations, and pseudotime trajectory of germ cells in human (from left to right). **B** Dot plot displaying cell type-specific marker genes and germ cell subtype-specific markers in human testis. **C** UMAP visualization showing the distribution of all cell types, major cell populations, and pseudotime trajectory of germ cells in pig (from left to right). **D** Dot plot displaying cell type-specific marker genes and germ cell subtype-specific markers in pig testis. **E** UMAP visualization showing the distribution of all cell types, major cell populations, and pseudotime trajectory of germ cells in mouse (from left to right). **F** Dot plot displaying cell type-specific marker genes and germ cell subtype-specific markers in mouse testis


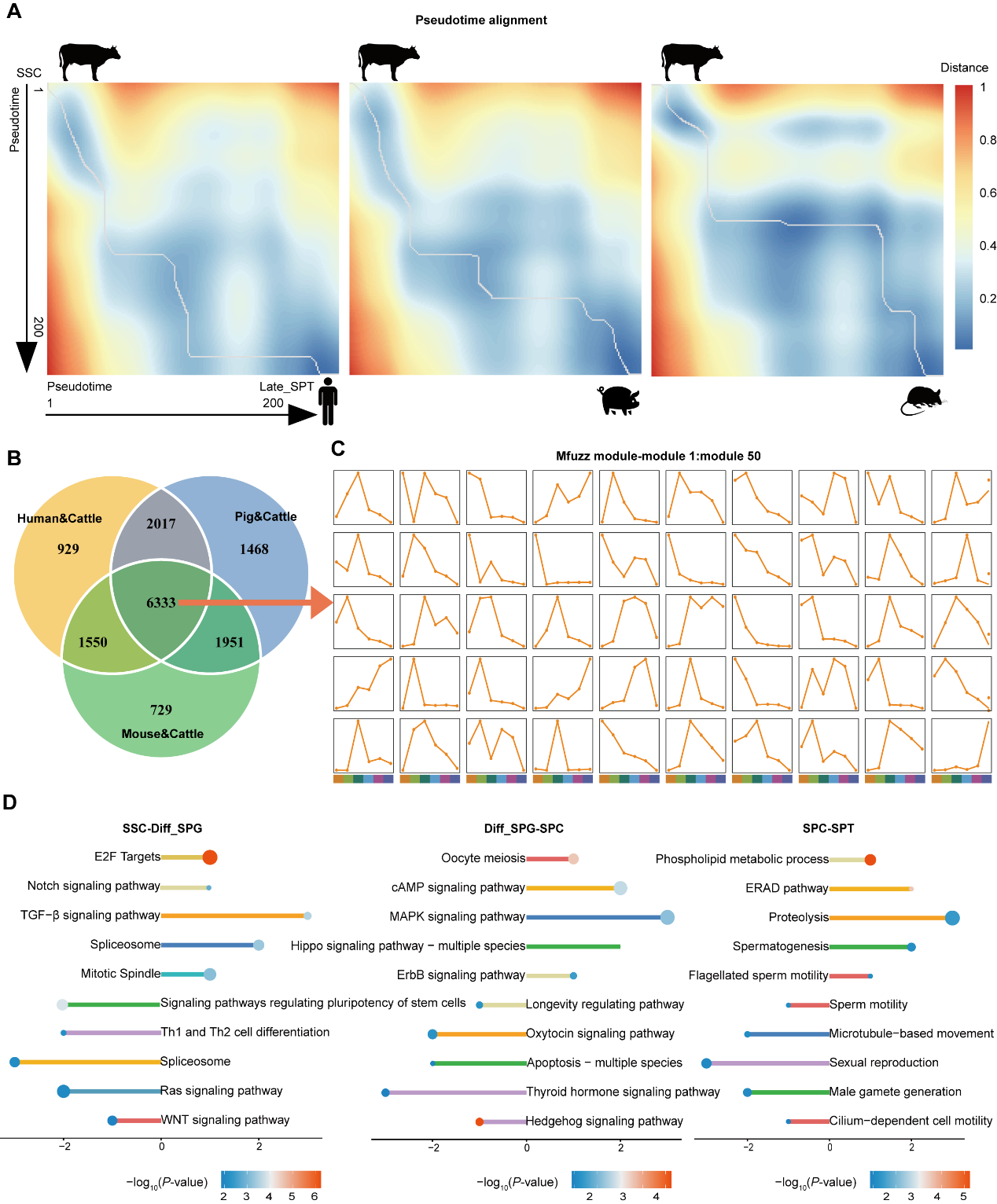


**Fig. S8** Comparative analysis of gene expression dynamics across humans, cattle, pigs, and mice during germ cell differentiation. **A** Correlation matrices depict the pseudotime heterochronicity between pairs of species (human and cattle, cattle and pig, and cattle and mouse, from left to right). These matrices compare 200 sequentially ordered centroids for each species, derived from their initial pseudotime assignments. The color intensity represents the extent of the distance. **B** Venn diagram illustrating the number of shared and unique genes among different species. **C** Cross-species mfuzz clustering of germ cell gene expression dynamics (human, pig, cattle and mouse) **D** Functional enrichment analysis of key pathways during SSC to Diff-SPG, Diff-SPG to SPC, and SPC to SPT transitions across species
