# Supplementary material for: Depicting the dynamic transcriptional and epigenetic landscape of testis development in pubertal Simmental cattle
Source: J Anim Sci Biotechnol. 2026 May 18;17:96. doi: 10.1186/s40104-026-01406-x (PMC13181952; doi:10.1186/s40104-026-01406-x)
Supplement: Supplementary file 2 — Additional file 2: Table S1. Information collection of Simmental cattle. Table S2. Simmental cattle information summary sheet. [file 40104_2026_1406_MOESM2_ESM.docx]

**Table S1** Information collection of Simmental cattle

| **Body weight** | **Age** | **Pedigree** | **Individual** |
| --- | --- | --- | --- |
| 45 kg | 4 days | father | 411217286 |
|  |  | mother | HX420002202123779 |
| 47 kg | 5 days | father | 22218119 |
|  |  | mother | HX42000220212111 |
| 211 kg | 5 months | father | 22218621 |
|  |  | mother | HX42000220212105 |
| 259 kg | 5 months | father | 22218621 |
|  |  | mother | HX15000820212304 |
| 342 kg | 7 months | father | 22218119 |
|  |  | mother | HX42000220212125 |
| 514 kg | 13 months | father | 22218119 |
|  |  | mother | HX42000220212200 |
| 489 kg | 14 months | father | 22218621 |
|  |  | mother | HX42000220212100 |

**Table S2** Simmental cattle information summary sheet

| **Samples** | **Age** | **Developmental stage** | **Assays** |
| --- | --- | --- | --- |
| 1 | 4 days | postnatal (PN) | scRNA-seq |
| 2 | 5 days | postnatal (PN) | sNucATAC-seq |
| 3 | 5 months | prepubertal (PP) | sNucATAC-seq |
| 4 | 5 months | prepubertal (PP1) | scRNA-seq |
| 5 | 7 months | prepubertal (PP2) | scRNA-seq |
| 6 | 13 months | pubertal (PUB) | sNucATAC-seq |
| 7 | 14 months | pubertal (PUB) | scRNA-seq |
